# GRP75 blocks hepatitis E virus infection by targeting HEV-ORF2 for degradation through chaperone-mediated autophagy and promoting IRF3 activation

**DOI:** 10.1128/jvi.01344-25

**Published:** 2025-12-17

**Authors:** Yajing Wang, Yafei Li, Rong Xu, Tong Yuan, Chenying Xu, Zhaobin Zhou, Cuiyu Ba, Qin Zhao, Chunyan Wu, Zhiru An, Xin Yin, Yonglin Yang, Yuchen Nan

**Affiliations:** 1 Department of Preventive Veterinary Medicine, College of Veterinary Medicine, Northwest A&F University 12469, Yangling, China; 2 College of Animal Science and Technology, Jiangxi Agricultural University 593244, Nanchang, Jiangxi, China; 3 State Key Laboratory for Animal Disease Control and Prevention, Harbin Veterinary Research Institute, Chinese Academy of Agricultural Sciences 687216 , Harbin, China; 4 Department of Infectious Diseases & Institute of hepatology, The Affiliated Taizhou People’s Hospital of Nanjing Medical University 372209 , Taizhou, Jiangsu Province, China; 5 Taizhou School of Clinical Medicine, Nanjing Medical University, Taizhou, Jiangsu Province, China; Wake Forest University School of Medicine, Winston-Salem, North Carolina, USA

**Keywords:** CMA, GRP75, hepatitis E virus (HEV), interferon, mitochondria, ORF2

## Abstract

Hepatitis E virus (HEV) is a viral hepatitis pathogen that poses a significant threat to global human health, representing a serious yet long-overlooked public health concern. In this study, we identified glucose-regulated protein 75 (GRP75) as an interaction partner of HEV-ORF2 using recombinant ORF2 truncation as bait. The substrate-binding domain of GRP75 interacted with HEV-ORF2 and inhibited HEV replication by facilitating HEV-ORF2 degradation. Further analysis revealed that HEV-ORF2 contains three KFERQ-like motifs, the key signature sequence required for chaperone-mediated autophagy (CMA). Our data demonstrated that GRP75-mediated degradation of HEV-ORF2 was heat-shock cognate protein 70 (HSC70)-dependent, although no direct interaction between HSC70 and ORF2 was detected. Instead, GRP75, together with HEV-ORF2 and HSC70, formed a complex that mediated CMA-dependent degradation of HEV-ORF2, whereas deletion of all three KFERQ-like motifs from ORF2 conferred resistance to such processes. Additionally, GRP75 blocked mitochondrial transport of HEV-ORF2, potentially mitigating ORF2’s function as an interferon (IFN) induction antagonist. Furthermore, GRP75 enhanced the interaction between mitochondrial antiviral signaling protein (MAVS) and TANK-binding kinase 1 (TBK1), promoting IFN-β production and ultimately inhibiting HEV infection. In conclusion, our findings identify GRP75 as a novel restriction factor for HEV infection and provide new insights into its role in CMA and antiviral innate immunity.

**IMPORTANCE:**

Due to the lack of an effective *in vitro* model, the viral-host interaction of HEV remains largely elusive. This study uncovers a novel mechanism by which GRP75 inhibits HEV infection. On one hand, the GRP75 protein facilitates the degradation of HEV-ORF2 through the lysosome-associated, chaperone-mediated autophagy by recognizing KFERQ-like motif presented on HEV-ORF2. On the other hand, GRP75 enhances the production of IFN-β by promoting interaction between MAVS and TBK1, thereby establishing an antiviral state and suppressing HEV infection. This research expands our current understanding of host resistance to HEV and provides a new function of GRP75, suggesting that GRP75 might be a novel antiviral factor against virus infection.

## INTRODUCTION

Hepatitis E virus (HEV) is a single-stranded, positive-sense RNA virus, categorized within the expanding *Hepeviridae* family (1). This family encompasses zoonotic, anthropotropic, and animal-restricted HEV species, as well as HEV-like viral isolates from various hosts (1). HEV viral particles exist in both non-enveloped and enveloped forms; the latter was recently defined as a quasi-enveloped viral form (2, 3). Initially, HEV is thought to be restricted to humans, causing a self-limiting hepatitis (4), with outbreaks predominantly reported in developing countries in Asia, Africa, and Central America. However, the discovery of novel HEV isolates in swine in 1997 suggested a broader host range and zoonotic potential for HEV (5). Currently, numerous sporadic cases have been documented in developed countries in Europe, the United States, and Japan as HEV’s host range continues to expand (6–8). Globally, HEV infects approximately 20 million individuals annually, with 3.3 million symptomatic cases and an estimated 70,000 deaths. Meanwhile, chronic HEV infection, HEV-related acute hepatic failure, and extrahepatic manifestations have been frequently reported (9–13), indicating a complex mechanism underlying HEV-related diseases. However, due to the lack of effective *in vitro* culture systems, critical aspects of HEV biology, such as its cellular receptors, host proteins required for infection, and cross-species transmission factors, remain poorly understood.

HEV contains a 7.2-kb mRNA-like genome that is capped and polyadenylated. To date, three well-recognized open-reading frames (ORFs) have been identified in all HEV isolates (14, 15), whereas an additional ORF4 has only been reported in HEV-1 (16). HEV-ORF1 encodes a non-structural polyprotein that functions as the viral replicase, directly translated from the viral genome (17). In contrast, HEV-ORF2 and ORF3, which partially or completely overlap, require translation from subgenomic RNA (18). HEV-ORF2 encodes the major capsid protein, whereas HEV-ORF3 encodes a multifunctional protein that is likely a class I viroporin and essential for virion release (19).

In mammalian HEV, the full-length ORF2 protein contains 660 amino acids (aa) and was initially identified as a capsid protein required for virion assembly (20). Sequence analysis indicates that the full-length ORF2 protein carries N-terminally linked glycans at three different asparagine (Asn) sites (aa137, 310, and 562), along with an endoplasmic reticulum (ER)-directing signal peptide in its N-terminus (14). The ER-directing signal peptide targets full-length ORF2 for subsequent glycosylation and secretion (21), whereas alternative translation initiated at an internal start codon (aa16 of the full-length ORF2) downstream of the signal peptide generates a shorter form of ORF2 protein, which is responsible for forming mature capsid protein associated with infectious HEV virions (21, 22). Thus, HEV-ORF2 exists in multiple functional forms rather than solely serving as a viral capsid protein (22).

Studies have categorized HEV-3 ORF2 products into three distinct forms: ORF2i (infectious, virion-associated form), ORF2g (glycosylated, secreted form), and ORF2c (cleaved, secreted form) (22). HEV-specific antibodies recognize all three forms, but only ORF2i is incorporated into infectious virions (22). Moreover, glycosylated full-length ORF2 forms a dimer with altered antigenicity relative to the HEV capsid protein, as predicted cell receptor-binding epitopes are lost (21). Consequently, the secreted full-length ORF2 protein does not block HEV cell entry but inhibits antibody-mediated neutralization (21). Among the three potential glycosylation sites of N1 (aa137), N2 (aa310), and N3 (aa562), only N1 and N3 are glycosylated in ORF2g/c, whereas ORF2i remains unglycosylated (22).

The HEV capsid is formed by unglycosylated ORF2 proteins (21, 22). Although recombinant HEV ORF2 protein lacking 111 and 52 amino acids at its N- and C-termini can form virus-like particles (VLPs) when expressed in insect cells (23, 24), the exact length of the ORF2 protein associated with the native HEV virion still requires further characterization. Both conformational and linear neutralizing epitopes have been identified in HEV capsid protein (25, 26), and a recombinant subunit vaccine based on a truncated HEV capsid protein has been licensed in China. Additionally, ORF2 functions as a potential IFN antagonist, forming a multiprotein complex with MAVS, TBK1, and IRF3 to inhibit IRF3 phosphorylation, effectively suppressing IFN-β production. The N-terminal domain of ORF2 inhibits IRF3 activation, with its Arg-rich motifs playing a critical role in this process. These findings provide new insights into HEV immune evasion mechanisms (27).

In this study, we demonstrate that GRP75 is a novel restriction factor for HEV infection. GRP75 suppresses HEV replication by inducing ORF2 degradation via chaperone-mediated autophagy (CMA). Additionally, GRP75 enhances the MAVS-TBK1 interaction, promoting IFN-β production and antiviral gene expression, thereby inhibiting HEV infection. These findings expand our understanding of host resistance to HEV and host-HEV interactions.

## RESULTS

### GRP75 is an interacting partner of the HEV-ORF2 protein

In our preliminary studies, we demonstrated that the truncated ORF2 protein (corresponding to the HEV-p239 portion of the subunit vaccine) from both swine HEV-4 and avian HEV could form VLPs when expressed in *Escherichia coli*. These VLPs resembled viral capsid proteins and were capable of binding host cells, making them suitable for identifying cellular interacting partners (28, 29). Based on this approach, we cloned the ORF2-p239 (K239) sequence from the HEV-3 KernowC1-p6 strain to identify potential cellular proteins interacting with ORF2 of HEV-3 KernowC1-p6 since it is the only cell-adapted HEV strain that could be cultured *in vitro* (30).

**Fig 1:**
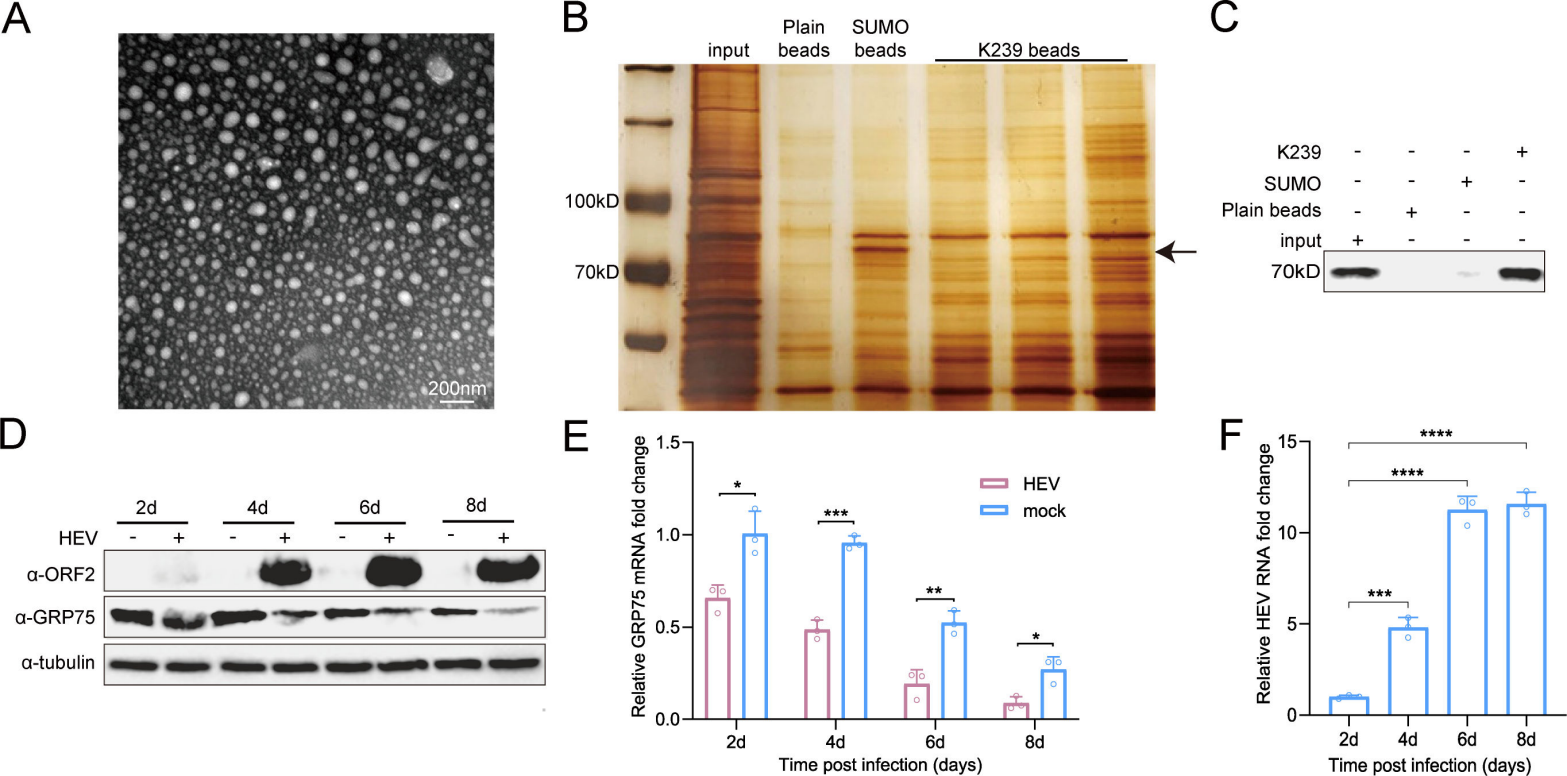
Identification of GRP75 as an HEV-ORF2-interacting protein. (**A**) Recombinant K239 protein forms virus-like particles (VLPs) resembling HEV capsids. Truncation of HEV-ORF2 forms VLP. The K239 protein was expressed in *E. coli*, refolded in PBS, and analyzed using electron microscopy (EM) to confirm VLP formation. Scale bar = 200 nm. (**B**). Identification of potential protein interactions with K239. The recombinant K239 protein was conjugated to CNBr-activated Sepharose 4B resin and incubated with plasma membrane extracts from S10-3 cells. SDS-PAGE and subsequent silver staining were performed to visualize potential interacting proteins. Plain CNBr-activated Sepharose 4B resin and recombinant SUMO protein-conjugated Sepharose 4B resin served as blank and irrelevant protein controls, respectively. (**C**) Western blot verification of GRP75 interaction with K239. K239-conjugated CNBr-activated Sepharose 4B resin was used to probe plasma membrane extracts from S10-3 cells. After SDS-PAGE, western blotting with an anti-GRP75 antibody was performed to confirm the presence of GRP75 in the pull-down complex. (**D**) HEV replication inhibits GRP75 expression in S10-3 cells. S10-3 cells were transfected with HEV-RNA (KernowC1-p6 strain), and samples were collected at various time points. Western blotting was performed to assess GRP75 protein levels over time. (**E**) Quantification of GRP75 mRNA in HEV-replicating S10-3 cells. Total RNA was extracted from S10-3 cells at different time points post-transfection using TRIzol reagent. The qPCR was performed to measure GRP75 mRNA expression, using GAPDH mRNA as an internal control. GRP75 mRNA levels from uninfected S10-3 cells collected at the same time points were included as controls. Data are presented as mean ± SD and analyzed using Student’s *t*-test. *, *P* < 0.05; **, *P* < 0.01; ***, *P* < 0.001; ns, not significant. All results are based on at least three independent biological replicates. (**F**) Quantification of HEV RNA in HEV-replicating S10-3 cells. Total RNA was extracted from S10-3 cells at different time points post-transfection using TRIzol reagent. Then, qPCR was performed to evaluate replication of HEV-RNA. Data are presented as mean ± SD and analyzed using Student’s *t*-test. ***, *P* < 0.001; ****, *P* < 0.0001.

As shown in Fig. 1A, recombinant K239 protein formed VLPs similar to those found in swine HEV-4 and avian HEV. To identify interacting partners, we conjugated the K239 protein to cyanogen bromide (CNBr)*-*activated Sepharose 4B beads and incubated the conjugate with plasma membrane proteins extracted from S10-3 cells. Silver staining revealed a distinct protein band above 70 kDa, which was subjected to mass spectrometry (Fig. 1B). The results identified GRP75 (also known as HSPA9) as a potential ORF2-interacting partner. To further confirm this interaction, we performed a pull-down assay using K239-conjugated CNBr-Sepharose 4B beads, followed by immunoblotting with a GRP75-specific antibody. This analysis confirmed the presence of GRP75 in the ORF2-bound protein complexes (Fig. 1C).

To investigate whether GRP75 expression is affected by HEV replication, we transfected S10-3 cells with *in vitro*-transcribed HEV-3 KernowC1-p6 RNA. As shown in Fig. 1D, ORF2 expression was detected at a low level as early as 2 days post-transfection and continually increased to a stable level after 4 days, which is consistent with HEV-RNA replication (Fig. 1F). On the contrary, although GRP75 expression in mock cells demonstrated a decreased expression as culturing time for S10-3 cells extended, interestingly, GRP75 protein level in HEV-RNA transfected cells showed an enhanced decline from day 2 at the same time point. In parallel, GRP75 mRNA level exhibited a similar trend, which showed a downward trend from day 2 onward (Fig. 1F). These findings suggest that HEV replication in S10-3 cells inhibits GRP75 expression, implying that GRP75 might play a potential role during HEV replication.

**Fig 2:**
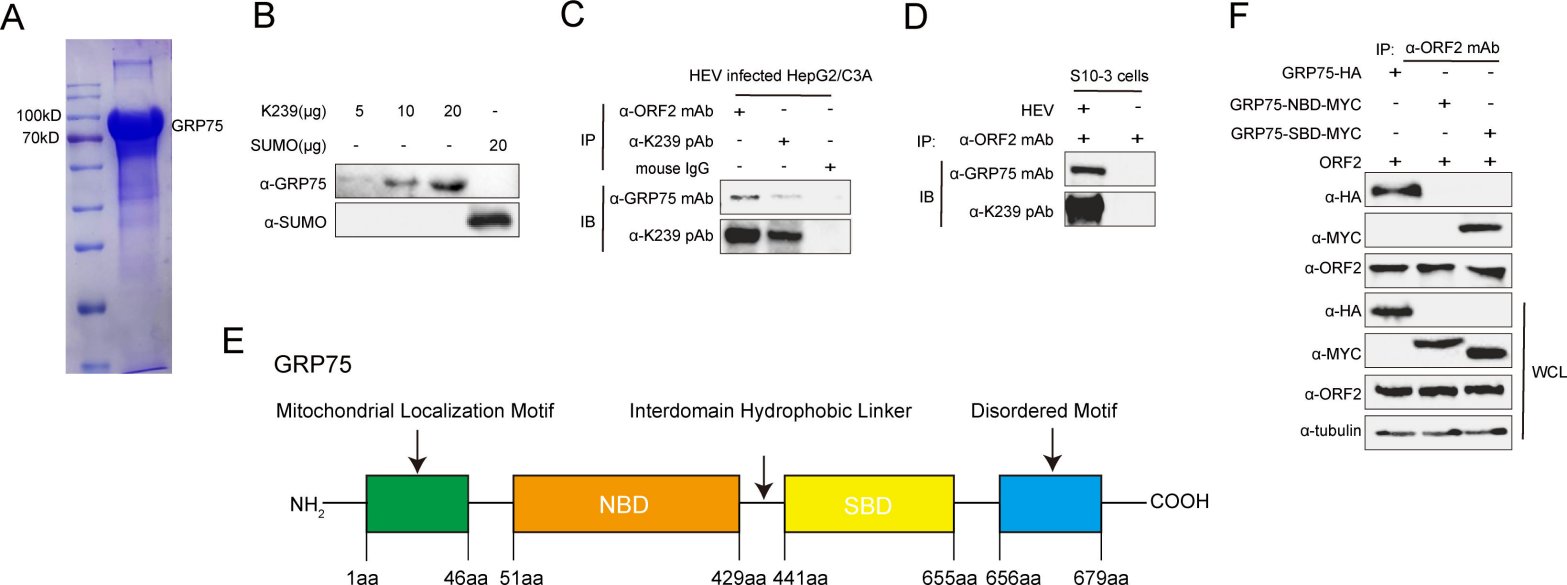
GRP75 interacts with HEV-ORF2 through its substrate-binding domain. (**A**) SDS-PAGE analysis for recombinant GRP75. Recombinant GRP75 was expressed from the *E. coli* system and refolded into PBS, then subjected to SDS-PAGE analysis. (**B**) Far-western blot assay for detecting the interaction between K239 and GRP75. Different doses of recombinant K239 protein were subjected to SDS-PAGE and transferred to a PVDF membrane. Next, the membrane was incubated with recombinant GRP75 protein expressed in *E. coli*, followed by detection using an anti-GRP75 antibody and corresponding secondary antibody to assess the binding between K239 and recombinant GRP75. Recombinant SUMO protein was included as an irrelevant control. (**C**) Co-immunoprecipitation (CoIP) analysis of the GRP75 and HEV-ORF2 interaction in HEV-infected HepG2/C3A cells. HepG2/C3A cells stably infected with HEV-3 KernowC1-p6 were lysed using NP-40 buffer and subjected to immunoprecipitation (IP) with an ORF2-specific monoclonal antibody (Mab) 2G8 to detect GRP75 within the ORF2 complex using a GRP75-specific antibody. Normal mouse IgG (mIgG) served as an antibody isotype control for IP. (**D**) CoIP assay for GRP75 and HEV-ORF2 interaction in HEV RNA-transfected S10-3 cells. S10-3 cells were transfected with HEV-3 KernowC1-p6 RNA and cultured for 7 days. Cells were then harvested for Co-IP analysis, following the same protocol as described above. Uninfected control cells were included for comparison. (**E**) Schematic illustration of GRP75 function domains. (**F**) The interaction of GRP75 with ORF2 depends on the substrate-binding domain (SBD) of GRP75. HEK-293T cells were transfected with plasmids encoding MYC-tagged full-length GRP75 (GRP75-MYC), nucleotide-binding domain (NBD) (NBD-MYC), or substrate binding domain (SBD-MYC), along with a plasmid encoding HEV-ORF2, for 48 h. Next, cells were lysed with NP-40 buffer, and proteins were immunoprecipitated using the ORF2-specific Mab-2G8, followed by western blotting using a MYC tag-specific monoclonal antibody to elucidate domain-specific interactions. The proteins from whole cell lysate (WCL) were probed to confirm the expression of the transfected plasmids.

To further validate the interaction between GRP75 and ORF2, we employed far-western blotting using recombinant GRP75 (Fig. 2A) and confirmed the direct binding of GRP75 to ORF2 *in vitro* (Fig. 2B). Additionally, immunoprecipitation (IP) assays in HEV-infected HepG2/C3A cells and S10-3 cells transfected with HEV RNA demonstrated that GRP75 interacted with HEV-ORF2 during viral infection in permissive cells (Fig. 2C and D), providing strong evidence that GRP75 is an interacting partner of HEV-ORF2 during HEV infection.

GRP75 belongs to the HSP70 chaperone family and contains distinct functional domains, including the nucleotide-binding domain (NBD) and the substrate-binding domain (SBD) (Fig. 2E), which bears ATPase activity and mediates protein-protein interactions, respectively. We next sought to determine which domain of GRP75 is responsible for ORF2 binding. To address this, plasmids encoding full-length, NBD, and SBD of GRP75 were constructed and fused with a MYC tag, followed by co-transfection with the ORF2-encoding plasmid into HEK-293T cells. The IP analysis revealed that ORF2 specifically interacted with the SBD of GRP75 (Fig. 2F). In summary, these results demonstrate that the SBD of GRP75 interacts with HEV-ORF2 and likely functions as its chaperone.

### GRP75 inhibits HEV infection in susceptible cells by targeting ORF2

**Fig 3:**
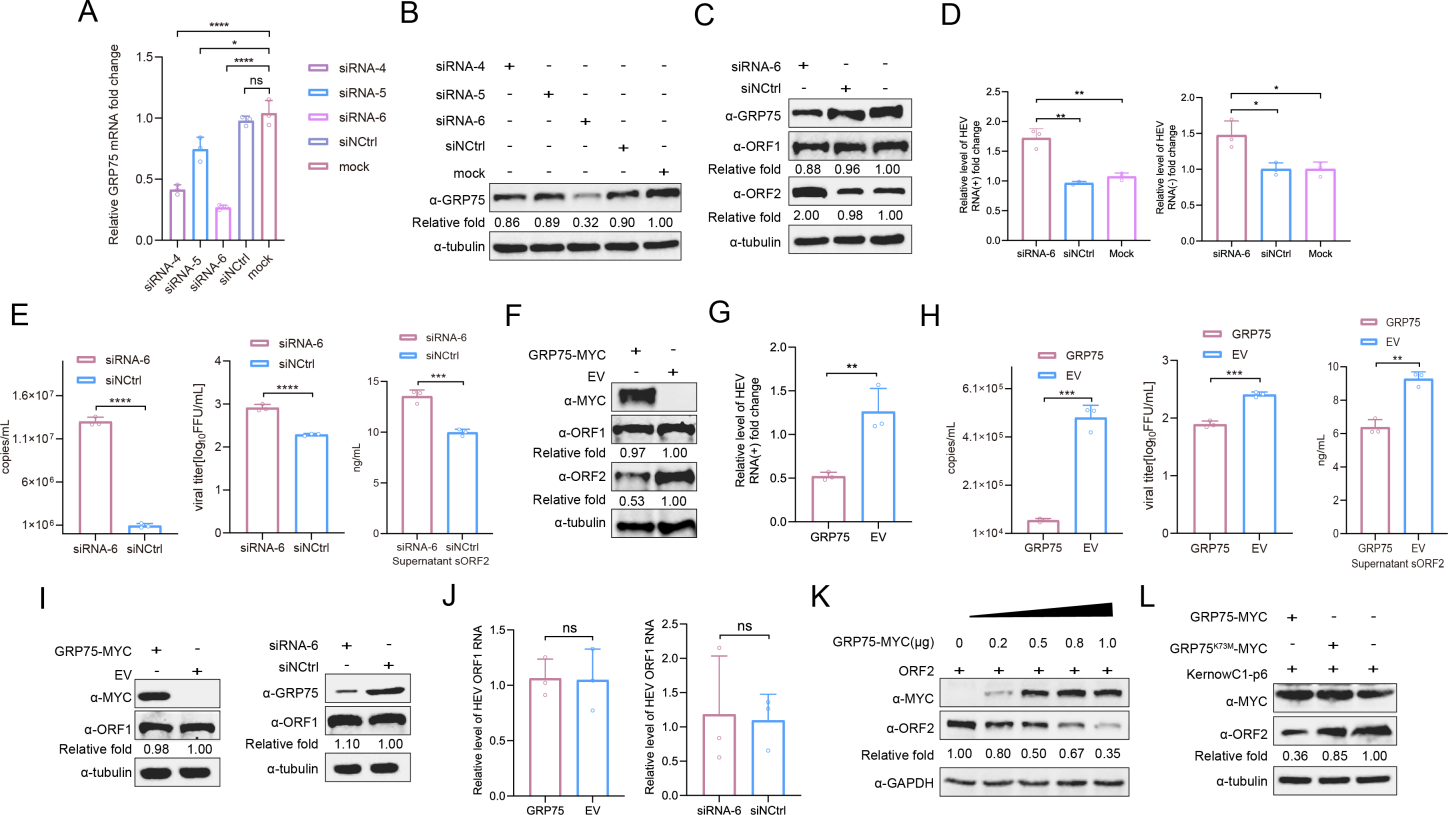
GRP75 inhibits HEV infection by targeting ORF2. (**A**) The qPCR validation of GRP75 knockdown using siRNA. S10-3 cells either GRP75-specific siRNA-4, 5, and 6 or control siRNA (siNCtrl). After 48 hours, the cells were harvested using TRIzol reagent. Next, qPCR was performed to quantify the mRNA level of GRP75. All data are presented as mean ± SD and were analyzed using Student’s *t*-test. ****, *P* < 0.0001. All results were derived from three independent biological replicates. (**B**) Western blotting validation of GRP75 knockdown using siRNA. S10-3 cells either GRP75-specific siRNA-4, 5, and 6 or control siRNA (siNCtrl). After 48 h, the cells were harvested for SDS-PAGE, followed by western blotting to assess GRP75 protein levels. Tubulin served as a loading control. (**C**) Knockdown of GRP75 increased HEV-ORF2 expression in HEV-infected cells. HepG2/C3A cells stably infected by HEV were transfected with either GRP75-specific siRNA-6 or control siRNA (siNCtrl). After 48 h, the cells were harvested for SDS-PAGE, followed by western blotting to assess GRP75, HEV-ORF1, and ORF2 protein levels. (**D**) Knockdown of GRP75 promotes HEV-RNA levels in HEV-replicating cells. S10-3 cells stably transfected by HEV were further transfected with either GRP75-specific siRNA or control siRNA, followed by RNA extraction using TRIzol reagent. The qPCR was performed to quantify both positive-strand (+) and negative-strand (−) HEV RNA levels. Untreated HEV-replicating S10-3 cells (MOCK control) served as a control. All data are presented as mean ± SD and were analyzed using Student’s *t*-test. *, *P* < 0.05; **, *P* < 0.01; ****, *P* < 0.0001; ns, not significant. All results were derived from three independent biological replicates. (**E**) Knockdown of GRP75 promotes the release of HEV virion and secreted ORF2 (sORF2). S10-3 cells stably transfected by HEV were further transfected with either GRP75-specific siRNA or control siRNA. Then, cell culture supernatant was harvested for qPCR to evaluate the HEV-RNA copy numbers (left panel), for virus titration in HepG2/C3A cells (middle panel), or for sandwich ELISA to evaluate secreted ORF2 (sORF2). All data are presented as mean ± SD and were analyzed using Student’s *t*-test. ***, *P* < 0.001; ****, *P* < 0.0001. All results were derived from three independent biological replicates. (**F**) Overexpression of GRP75 inhibits HEV-ORF2 protein level in HEV-replicating cells. S10-3 cells stably transfected with HEV RNA were further transfected with either a plasmid encoding GRP75-MYC or an EV. After 48 h, the cells were harvested for western blotting to assess GRP75, ORF1, and HEV-ORF2 protein levels. Tubulin served as a loading control. (**G**) GRP75 inhibits HEV-RNA levels in HEV-replicating cells. S10-3 cells stably infected by HEV were further transfected with either a plasmid encoding GRP75-MYC or EV. After 48 h, total RNA was extracted, and qPCR was performed to quantify total HEV RNA levels. GAPDH expression from the same cDNA was used as an internal control. All data are presented as mean ± SD and were analyzed using Student’s *t*-test. **, *P* < 0.01; ****, *P* < 0.0001; ns, not significant. All results were based on three independent biological replicates. (**H**) GRP75 inhibits releasing of HEV virion and secreted ORF2 (sORF2). S10-3 cells stably infected by HEV were further transfected with either a plasmid encoding GRP75-MYC or empty vector (EV). After 48 h, the cell culture supernatant was harvested for qPCR to evaluate the HEV-RNA copies numbers (left panel), for virus titration in HepG2/C3A cells (middle panel), or for sandwich ELISA to evaluate secreted ORF2 (sORF2). All data are presented as mean ± SD and were analyzed using Student’s *t*-test. **, *P* < 0.01; ***, *P* < 0.001. All results were derived from three independent biological replicates. (**I**) GRP75 did not affect HEV-ORF1 level in HEV-zsGreen replicon system. S10-3 cells transfected with HEV-zsGreen replicon RNA were further transfected with either GRP75 encoding plasmid (left panel) or GRP75-specific siRNA-6 (right panel). After 48 h, the cells were harvested for SDS-PAGE, followed by western blotting to assess GRP75 and HEV-ORF1 and ORF2 protein levels. Cells transfected with empty vector (EV) or control siRNA (siNCtrl) were included as controls, respectively. (**J**) GRP75 did not affect HEV-RNA level in HEV-zsGreen replicon system. S10-3 cells transfected with HEV-zsGreen replicon RNA were further transfected with either GRP75 encoding plasmid (left panel) or GRP75-specific siRNA-6 (right panel). Cells transfected with empty vector (EV) or control siRNA (siNCtrl) were included as controls, respectively. After 48 hours, total RNA was extracted, and qPCR was performed to quantify total HEV RNA levels. GAPDH expression from the same cDNA was used as an internal control. All data are presented as mean ± SD and were analyzed using Student’s *t*-test. ns, not significant. (**K**) GRP75 inhibits HEV-ORF2 expression in a dose-dependent manner. HEK-293T cells were transfected with the HEV-ORF2-encoding plasmid along with increasing amounts of a GRP75-encoding plasmid. After 48 h, the cells were harvested for western blotting to assess GRP75 and HEV-ORF2 expression levels. GAPDH served as a loading control to normalize total protein input. (**L**) Inhibition of HEV-ORF2 protein level requires the ATPase activity of GRP75. S10-3 cells stably replicating HEV RNA were transfected with plasmids encoding MYC-tagged wild-type GRP75 (WT GRP75), an ATPase-deficient mutant (GRP75^K73M^), or EV for 48 h. Western blotting was performed to examine ORF2 protein expression levels. Tubulin served as a loading control.

Heat-shock proteins (HSPs) constitute a large family of chaperones present in most eukaryotes and bacteria, playing essential roles in protein folding and cellular protection from stress-induced damage, including that caused by viral infections (31). To further explore the role GRP75 played during HEV infection, we conducted siRNA-mediated knockdown of GRP75 and transfection of S10-3 cells with GRP75-specific siRNA revealing that siRNA-6 significantly reduced GRP75 protein expression, whereas the negative control siRNA (siNCtrl) had no effect (Fig. 3A and B). Next, HEV-replicating cells were transfected with GRP75-specific siRNA-6 and monitored for viral replication. The results showed that GRP75 knockdown significantly increased HEV-ORF2 protein level in HEV-replicating cells, whereas the HEV-ORF1 protein level was not affected (Fig. 3C). Meanwhile, qPCR analysis of both positive-HEV-RNA (HEV-RNA(+)) and negative-strand HEV-RNA (HEV-RNA(−)) demonstrated that siRNA-mediated knockdown of GRP75 increased HEV-RNA level in infected cells, whereas control siRNA transfection had a minimal effect (Fig. 3D). Moreover, the release of infectious HEV virion and secretion of ORF2 in cell culture supernatant were investigated as well. As shown in Fig. 3E, GRP75 knockdown significantly enhanced the release of infectious HEV particles as well as the secretion of ORF2 in supernatant. On the contrary, overexpression of GRP75 in HEV-replicating cells resulted in a significant reduction in ORF2 protein and viral RNA (Fig. 3F and G), whereas the HEV-ORF1 protein level was similar in both groups. Similarly, a significant reduction of infectious virion and secreted ORF2 in the supernatant was observed in HEV-replicating cells with GRP75 overexpression (Fig. 3H).

Since HEV-ORF1 acts as HEV replicase required for viral RNA replication, it is notable that intracellular HEV-RNA level correlated with the changes of ORF2 under the GRP75 knockdown or overexpression condition, while unrelated with ORF1 protein level. To investigate whether GRP75 plays a role in HEV-RNA replication, a HEV-3 KernowC1-p6 strain-based replicon system with replacement of ORF2/3 by a zsGreen ORF (KernowC1-p6-zsGreen) was employed. In the HEV-replicon system devoid of ORF2/3, overexpression or knockdown of GRP75 demonstrated the minimum effect on HEV-ORF1 protein level and HEV-replicon RNA level (Fig. 3I and J). Therefore, these data imply that GRP75 inhibits HEV infection mainly through targeting HEV-ORF2 by direct interaction and inhibition of ORF2 expression in HEV-replicating cells but did not participate in HEV-RNA replication process. The increased or decreased HEV-RNA level in wild-type HEV replicating cells after knockdown or overexpression of GRP75 might be a consequence caused by changed HEV-ORF2 protein levels, which resulted in alteration of HEV virion assembly, release, and infection of fresh cells.

To further validate this hypothesis of HEV-ORF2 as the direct target of GRP75, we employed a transient overexpression model, in which co-transfection of GRP75 and HEV-ORF2 led to a significant reduction of HEV-ORF2 protein level (Fig. 3K). This effect was correlated with the amount of GRP75 plasmid transfected into the cells. Meanwhile, to examine the role of GRP75’s chaperone activity on the inhibition of ORF2 protein level, we constructed an ATPase-deficient mutant of GRP75 (GRP75^K73M^) and co-transfected it with ORF2. The results showed that GRP75^K73M^ caused a minimal reduction in ORF2 level (Fig. 3L), indicating that the chaperone function of GRP75 is essential for its inhibition of ORF2 expression.

**Fig 4:**
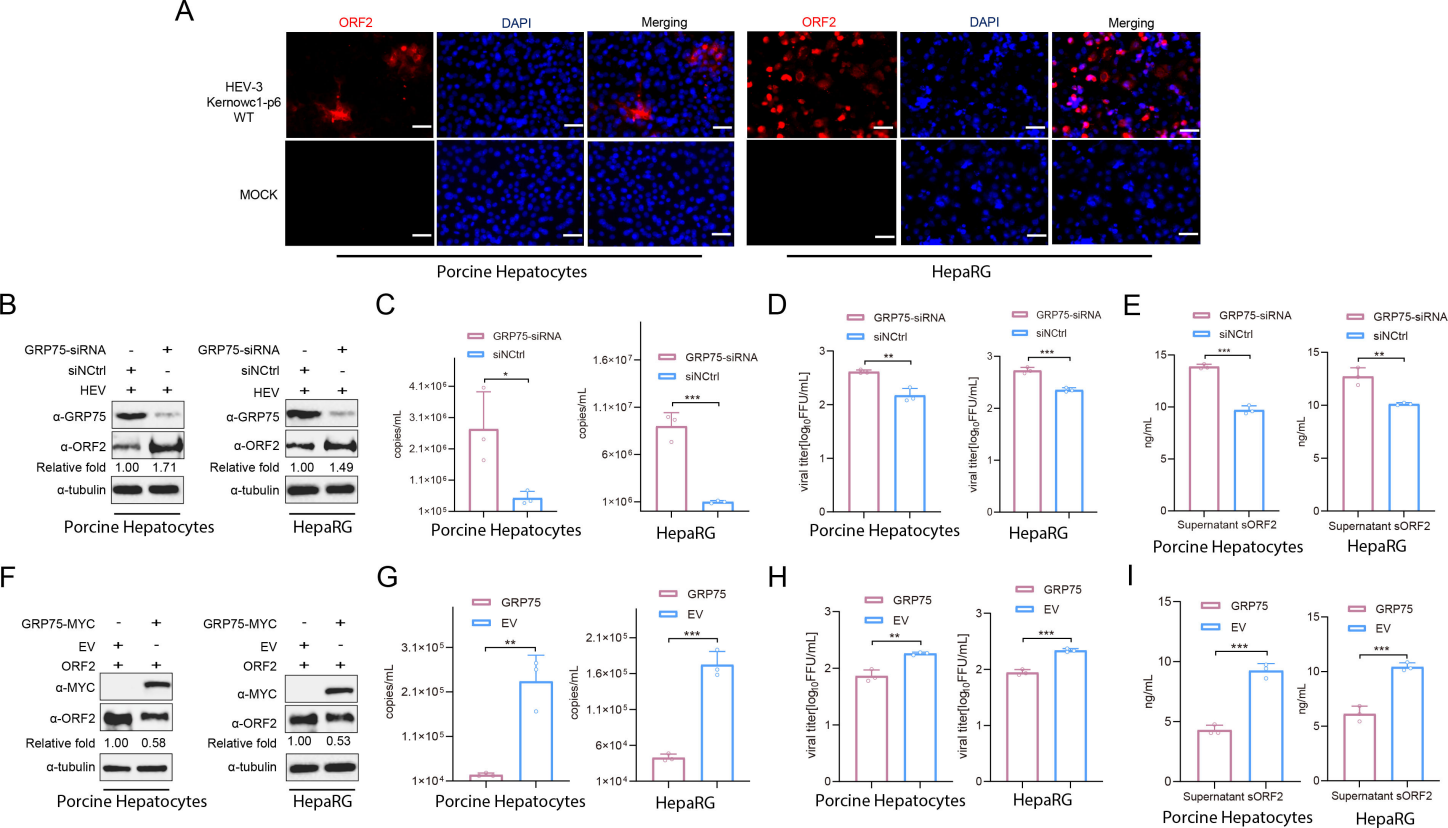
GRP75 inhibits HEV infection in primary porcine hepatocyte and HepaRG cells. (**A**) IFA examination of HEV replication in primary porcine hepatocyte and HepaRG cells. Porcine hepatocyte and HepaRG cells were transfected with HEV-RNA for 7 days; then, the cells were fixed and stained for ORF2 protein using ORF2-specific Mab-2G8, followed by observation under fluorescence microscopy. Scale bar = 100 μm. (**B**) Knockdown of GRP75 promotes HEV infection in primary porcine hepatocyte and HepaRG cells. Porcine hepatocyte (left panel) and HepaRG cells (right panel) were transfected with HEV-RNA for 7 days; then, the cells were further transfected with siRNA targeting porcine GRP75 (left panel) and human GRP75 (right panel). After 48 h post-siRNA transfection, the cells were harvested for western blotting to assess GRP75 and HEV-ORF2 expression levels. Tubulin was served as a loading control to normalize total protein input. (**C**) Knockdown of GRP75 promotes virion release of HEV from infected cells. HEV-RNA transfected porcine hepatocyte (left panel) and HepaRG cells (right panel) were further transfected with corresponding GRP75 siRNA for 48 h. Then, the cell culture supernatant was harvested for qPCR to quantify HEV-RNA copies. (**D**) Knockdown of GRP75 increases infectious viral titer of HEV in cell culture supernatant. HEV-RNA transfected porcine hepatocyte (left panel) and HepaRG cells (right panel) were further transfected with corresponding GRP75 siRNA for 48 h. Then, the cell culture supernatant was harvested for the titration of infectious HEV virion in HepG2/C3A cells. (**E**) Knockdown of GRP75 promotes secretion of ORF2. HEV-RNA transfected porcine hepatocyte (left panel) and HepaRG cells (right panel) were further transfected with corresponding GRP75 siRNA for 48 h. Then, the cell culture supernatant was harvested for sandwich ELISA to evaluate secreted ORF2 (sORF2). (**F**) Overexpression of GRP75 inhibits HEV infection in primary porcine hepatocyte and HepaRG cells. Porcine hepatocyte (left panel) and HepaRG cells (right panel) were transfected with HEV-RNA for 7 days; then, the cells were further transfected with plasmids encoding MYC tagged porcine GRP75 (left panel) and human GRP75 (right panel). After 48 h post-plasmid transfection, the cells were harvested for western blotting to assess GRP75-MYC and HEV-ORF2 expression levels. Tubulin was served as a loading control to normalize total protein input. (**G**) Overexpression of GRP75 inhibits virion release of HEV from infected cells. HEV-RNA transfected porcine hepatocyte (left panel), and HepaRG cells (right panel) were further transfected with plasmids encoding corresponding GRP75s. Then, the cell culture supernatant was harvested for titration in HepG2/C3A cells for infectious viral particles. (**H**) Overexpression of GRP75 inhibits infectious viral titer of HEV in cell culture supernatant. HEV-RNA-transfected porcine hepatocyte (left panel) and HepaRG cells (right panel) were further transfected with plasmids encoding corresponding GRP75s. Then, the cell culture supernatant was harvested for titration in HepG2/C3A cells for infectious viral particles. (**I**) Overexpression of GRP75 inhibits secretion of ORF2. HEV-RNA-transfected porcine hepatocyte (left panel) and HepaRG cells (right panel) were further transfected with plasmids encoding corresponding GRP75s. Then, the cell culture supernatant was harvested for sandwich ELISA to evaluate secreted ORF2 (sORF2). All data above are presented as mean ± SD and were analyzed using Student’s *t*-test. *, *P* < 0.05; **, *P* < 0.01; ***, *P* < 0.001. All results were derived from three independent biological replicates.

Since S10-3 cells are a hepatic carcinoma-derived cell line, to validate our result, primary porcine hepatocyte and HepaRG cells, an immortalized hepatic cell line that retains characteristics of primary human hepatocytes, were used for the validation of the above findings since primary human hepatocytes are hard to obtain. As demonstrated in Fig. 4A, primary porcine hepatocytes and HepaRG cells were permissive for HEV-3 KernowC1-p6 strain after HEV-RNA transfection and positive for HEV-ORF2 protein when examined using an immunofluorescence assay. In both cells, knockdown of GRP75 (porcine GRP75-specific siRNA was used for porcine hepatocyte) increased intracellular HEV-ORF2 level (Fig. 4B), promoted release of infectious HEV virion in supernatant (Fig. 4C and D), and enhanced the secretion of ORF2 (sORF2) protein (Fig. 4E). On the contrary, overexpression of GRP75 (porcine GRP75-encoding plasmid was used for porcine hepatocyte) not only inhibited HEV-ORF2 protein level in both cells (Fig. 4F) but also blocked the release of infectious HEV virion (Fig. 4G and H) and ORF2 secretion (Fig. 4I). Collectively, these results suggest that the interaction between HEV-ORF2 and GRP75 results in the suppression of ORF2 expression in HEV-replicating cells in a GRP75 chaperone activity-dependent manner.

### GRP75 promotes lysosomal degradation of HEV-ORF2

**Fig 5:**
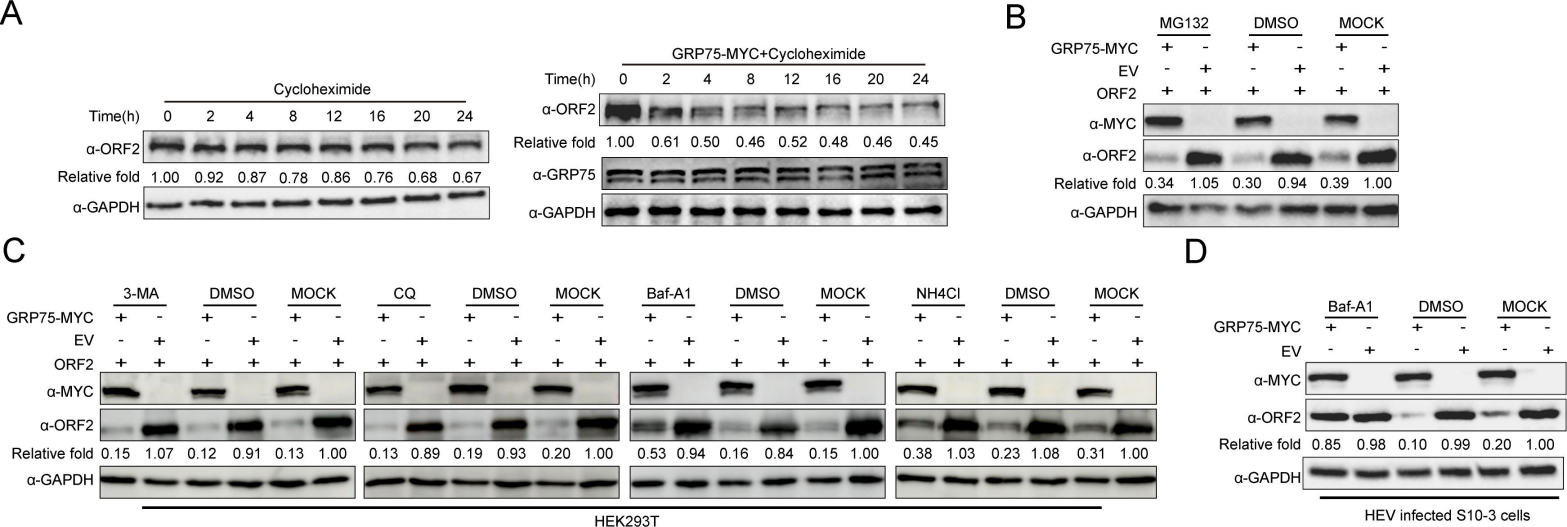
GRP75 promotes ORF2 degradation via a lysosome-dependent pathway. (**A**) GRP75 reduces the half-life of ORF2. HEV-replicating S10-3 cells were transfected with either empty vector (EV, left panel) or a GRP75-encoding plasmid (right panel) before treatment with cycloheximide (CHX, 10 μg) to inhibit global protein translation. Cells were harvested at 0, 2, 4, 8, 12, 16, 20, and 24 h post-CHX treatment for western blot analysis to determine the half-life of ORF2. GAPDH served as a loading control to normalize total protein input. (**B**) Degradation of HEV-ORF2 is not dependent on the ubiquitin-proteasome pathway. HEK-293T cells were transfected with either an EV or a GRP75-encoding plasmid, along with an ORF2-encoding plasmid, and incubated for 36 h. Next, the proteasome inhibitor MG132 (10 μM) was added, followed by incubation for an additional 16 h before cell harvest. Western blotting was performed to assess HEV-ORF2 protein levels, with GAPDH serving as a loading control. Cells treated with DMSO alone served as a solvent control. (**C**) Rescue of ORF2 protein levels depends on lysosome inhibitors. HEK-293T cells were transfected with either EV or a GRP75-encoding plasmid, along with an ORF2-encoding plasmid, and incubated for 36 hours. Cells were then treated with 3-MA (5 mM), CQ (50 μM), Baf-A1 (200 nM), and NH_4_Cl (15 mM) for 16 h before collection for western blot analysis. ORF2 expression levels were assessed to evaluate potential rescue effects, with GAPDH used as a loading control. Cells treated with DMSO alone were included as a solvent control. (**D**) Lysosome inhibitors rescue ORF2 protein in HEV-replication cells after GRP75 overexpression. S10-3 cells stably transfected with HEV RNA were further transfected with either a plasmid encoding GRP75-MYC or empty vector (EV) for 48 h. Cells were then treated with Baf-A1 (200nM) for another 16 h before collection for western blot analysis. ORF2 expression levels were assessed to evaluate potential rescue effects, with GAPDH used as a loading control. Cells treated with DMSO alone were included as a solvent control.

Since the above results suggest that GRP75 interacts with HEV-ORF2 and reduces HEV-ORF2 protein level in both HEV-replicating cells and ORF2 plasmid-transfected cells, it may imply that GRP75 promotes the degradation of HEV-ORF2 since GRP75 is not a protease. To confirm this hypothesis, we evaluated the half-life of ORF2 protein in the presence or absence of GRP75 by treating cells with Cycloheximide (CHX) to block universal protein translation. The results showed that the half-life of ORF2 exceeded 24 h in cells transfected with the ORF2-encoding plasmid alone (Fig. 5A). However, co-transfection of GRP75 significantly reduced the ORF2 half-life to 8 h (Fig. 5B), indicating that GRP75 accelerates HEV-ORF2 degradation.

To further identify specific protein degradation pathways contributing to the half-life reduction of ORF2 protein when co-expressed with GRP75, cells co-transfected with GRP75 and HEV-ORF2 were treated with inhibitors targeting different protein degradation pathways, including MG132 (ubiquitin-proteasome system inhibitor) and inhibitors of various stages of autophagy (3-MA, CQ, Baf-A1, and NH_4_Cl). Our results demonstrated that treatment with MG132, 3-MA, or CQ failed to restore ORF2 protein levels when co-expressed with GRP75 (Fig. 5B and C), indicating that GRP75-mediated ORF2 degradation does not depend on the ubiquitin-proteasome system or macroautophagy. However, treatment with Baf-A1, an autolysosome acidification inhibitor, partially rescued ORF2 protein level in the presence of GRP75, whereas NH_4_Cl had a weaker effect on ORF2 restoration than Baf-A1, implying GRP75-mediated degradation of HEV-ORF2 occurs through the lysosomal pathway, but not in a macroautophagy-dependent manner. Meanwhile, treatment of HEV-replicating S10-3 cells with Baf-A1 also resulted in partial restoration of ORF2 protein levels in the presence of GRP75 (Fig. 5D). Taken together, these findings suggest that GRP75-mediated protein half-life reduction of HEV-ORF2 occurs via the lysosomal pathway but independent of the ubiquitin-proteasome system and macroautophagy.

### GRP75-mediated degradation of HEV-ORF2 is CMA- and heat-shock cognate protein 70 (HSC70)-dependent

**Fig 6:**
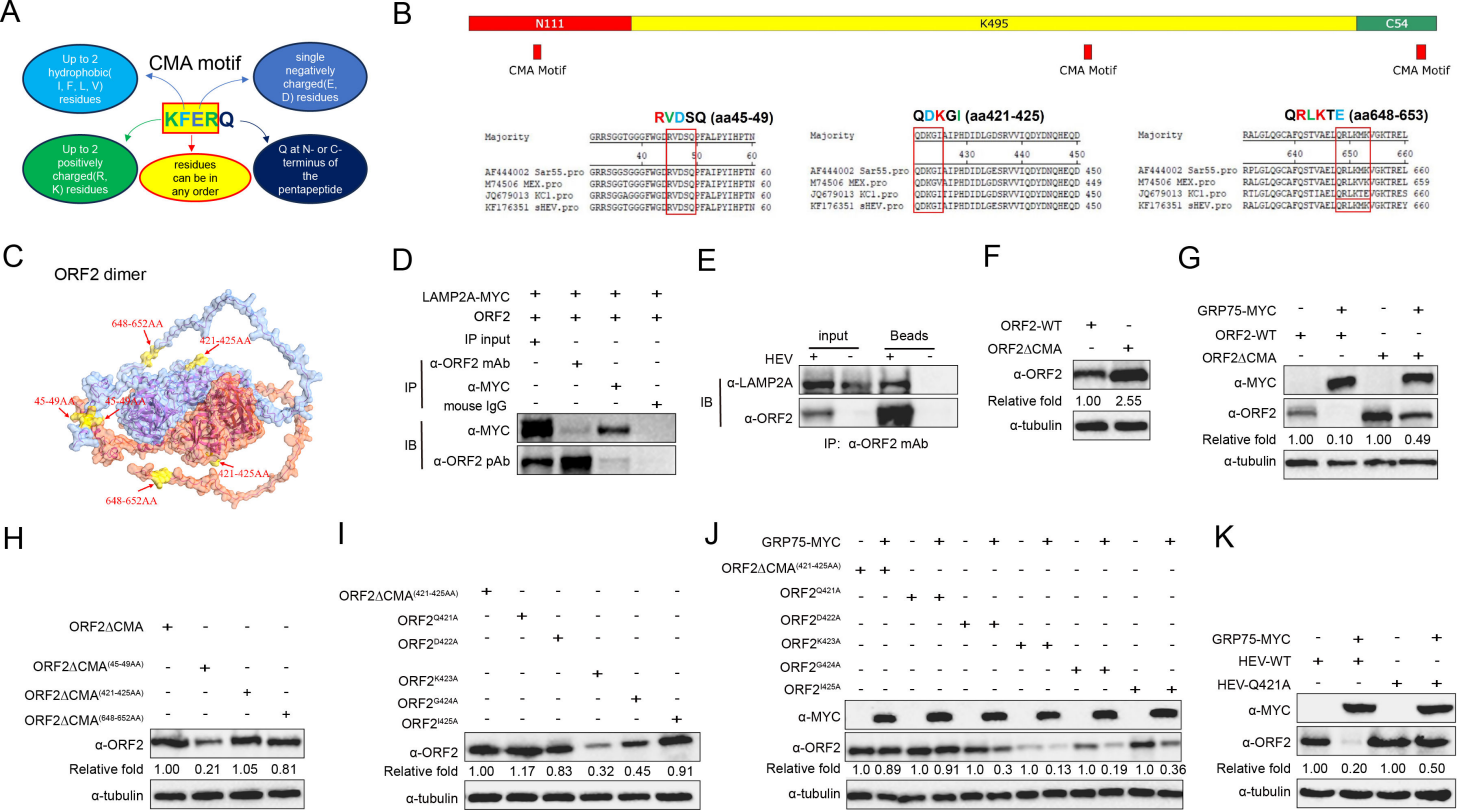
GRP75-mediated degradation of ORF2 depends on the KFERQ-like motif of ORF2. (**A**) Illustration of the KFERQ sequence standard as a motif for CMA. (**B**) Schematic illustration of the KFERQ-like motif in ORF2. The ORF2 sequences from all four HEV genotypes were compared and analyzed for the presence of potential CMA motifs using KFERQ finder V0.8. (**C**) Alphafold prediction for locations of KFERQ-like motif in HEV-ORF2 dimer from KernowC1-p6 strain. (**D**) Co-IP assay for ORF2-LAMP2A interaction in HEV-replicating S10-3 cells. S10-3 cells stably transfected with HEV-3 KernowC1-p6 RNA were lysed using NP-40 buffer, and ORF2-specific Mab-2G8 was used for IP. Western blotting was performed using a LAMP2A-specific monoclonal antibody to detect potential interactions. (**E**) Co-immunoprecipitation (Co-IP) assay for ORF2 interaction with LAMP2A in HEK-293T cells. HEK-293T cells were transfected with MYC-tagged LAMP2A (LAMP2A-MYC) and an HEV-ORF2-encoding plasmid for 48 h. Cells were lysed using NP-40 buffer, and immunoprecipitation (IP) was performed using MYC-specific and ORF2-specific monoclonal antibodies (Mab-2G8). The immunoprecipitated complexes were analyzed by western blot using MYC-specific monoclonal antibodies (Mab) for detecting LAMP2A and ORF2-specific polyclonal antibodies (pAb) for detecting ORF2, respectively. (**F**) Deletion of the KFERQ-like motif in ORF2 rescues ORF2 expression. HEK-293T cells were co-transfected with wild-type ORF2 (WT ORF2) or a KFERQ-like motif-deleted ORF2 mutant (ORF2ΔCMA). After 48 h, the cells were harvested for western blot analysis to assess ORF2 protein levels. Tubulin served as a loading control. (**G**) Deletion of the all CMA motif in ORF2 conferred resistance to GRP75-mediated degradation. HEK-293T cells were co-transfected with a GRP75-MYC-encoding plasmid or empty vector (EV), along with either wild-type ORF2 (WT ORF2) or a KFERQ-like motif-deleted ORF2 mutant (ORF2ΔCMA). After 48 h, the cells were harvested for western blot analysis to assess ORF2 protein levels. Tubulin served as a loading control. (**H**) KFERQ-like motif in aa421-425 contributed more role for the degradation of ORF2. HEK-293T cells were transfected with a plasmid encoding a complete KFERQ-like motif-deleted ORF2 mutant (ORF2ΔCMA), or an ORF2 mutant bearing the deletion of a different KFERQ-like motif (ORF2ΔCMA^45-49A^, ORF2ΔCMA^421-425A^, and ORF2ΔCMA^648-653A^). After 48 h, the cells were harvested for western blot analysis to assess ORF2 protein levels. Tubulin served as a loading control. (**I**) Screening for the key aa residue of the second KFERQ-like motif in aa421-425. HEK-293T cells were transfected with plasmids encoding deletion of KFERQ-like motif in aa421-425 in ORF2 mutant (ORF2ΔCMA^421-425A^), or ORF2 mutant bearing point mutation in KFERQ-like motif in aa421-425 (ORF2^Q421A^, ORF2,^D422A^ ORF2^K423A^,^G424A^ and ORF2^I425A^). After 48 h, the cells were harvested for western blot analysis to assess ORF2 protein levels. Tubulin served as a loading control. (**J**) Q421A mutation of ORF2 confers resistance to GRP75-mediated degradation through CMA. HEK-293T cells were co-transfected with a GRP75-MYC-encoding plasmid, along with plasmids encoding deletion of KFERQ-like motif in aa421-425 in ORF2 mutant (ORF2ΔCMA^421-425A^), or ORF2 mutants bearing single point mutation in aa421-425 (ORF2^Q421A^, ORF2^D422A^ ORF2^K423A^ ORF2^G424A^, and ORF2^I425A^). After 48 h, the cells were harvested for western blot analysis to assess ORF2 protein levels. Tubulin served as a loading control. (**K**) HEV bearing ORF2 Q421A confers resistance to GRP75-mediated degradation through CMA in HEV-replicating S10-3 cells. S10-3 cells transfected RNA of wild type HEV-3 KernowC1-p6 (HEV-WT) or HEV-3 KernowC1-p6 bearing Q421A mutation in ORF2 (HEV-Q421A) for 7days. Then, the cells were transfected with GRP75-MYC-encoding plasmid or empty vector (EV) for 48 h before the cells were harvested for western blot analysis to assess ORF2 protein levels. Tubulin served as a loading control.

Autophagy is classified into three primary forms: macroautophagy, microautophagy, and CMA (32, 33). Among these pathways, CMA is characterized by its substrate specificity through targeted degradation of proteins containing a KFERQ motif (Fig. 6A) (34, 35). Since our results indicated that GPR75 promoted lysosomal degradation of HEV-ORF2 independent of macroautophagy (a process requiring autophagosomal-lysosomal fusion), we analyzed the amino acid sequences of ORF2 from different HEV genotypes and identified three potential KFERQ-like motifs, with the first and the second motifs being highly conserved across major HEV genotypes (Fig. 5B). Moreover, by employing AlphaFold-based modeling for 3D structure of full-length ORF2 protein dimer, the locations of 3 potential KFERQ-like motifs in ORF2 dimer were presented as Fig. 6C. During CMA, cytoplasmic proteins containing a KFERQ-like motif bind to chaperone proteins; then, the resulting complexes are translocated to the lysosomal membrane. Once recognized by lysosome-associated membrane protein 2A (LAMP2A) on the lysosomal surface, the complexes are transported inside the lysosome for degradation (36, 37). To confirm whether ORF2 degradation occurs via the CMA pathway, we performed Co-IP analysis for detecting the interaction between ORF2 and LAMP2A, and a specific association between ORF2 and LAMP2A was revealed in both HEK-293T cells overexpressing ORF2 and LAMP2A (Fig. 5D), as well as HEV-replicating S10-3 cells (Fig. 5E).

To further verify whether GRP75-mediated degradation of ORF2 depends on the KFERQ-like motif, an HEV-ORF2 mutant lacking all three KFERQ-like motifs (ORF2ΔCMA) was constructed and compared with wild-type ORF2. In HEK-293T cells, ORF2ΔCMA-expressing cells displayed a significant elevation of ORF2 protein level compared to wild-type ORF2-expressing cells (Fig. 5F). Meanwhile, when co-transfected with GRP75, deletion of the KFERQ-like motifs in ORF2 protein conferred a partial resistance (50% reduction) to GRP75-mediated degradation when compared with cells only expressing ORF2ΔCMA (Fig. 5G), whereas co-transfection of GRP75 with wild-type ORF2 led to a 90% reduction of ORF2 protein level compared with cells expressing wild-type ORF2 alone (Fig. 5G). These data further indicate that GRP75-mediated ORF2 degradation is CMA-dependent.

Since three potential KFERQ-like motifs were presented in ORF2 and two of them (aa45-49 and aa421-425) were highly conserved across different HEV genotypes, we generated three mutants bearing deletion of individual motif to identify the crucial one. As shown in Fig. 6H, deletion of the second motif (aa 421–425) and the third motif (aa 648–653) resulted in a significant elevation of ORF2 protein level, in which deletion of the second motif restored more ORF2 protein level than the third one. Since the second motif was conserved in all 4 HEV genotypes, point mutation by mutagenesis assay was introduced into the second KFERQ-like motif, and the result demonstrated that expression level of ORF2^Q421A^ mutant was similar to ORF2ΔCMA^(421-425aa)^ (Fig. 6I). To further confirm that Q421 of ORF2 is the key residue for GRP75-mediated degradation of HEV-ORF2, all these mutants were co-transfected into HEK-293T cells with GRP75, and the result indicated ORF2^Q421A^ mutant conferred the highest resistance of GRP75-mediated degradation similar to that of ORF2ΔCMA^(421-425aa)^ (Fig. 6J). Additionally, by employing the reverse genetic system for HEV3-KernowC1-p6, a HEV mutant bearing ORF2^Q421A^ mutation was rescued and compared with wild-type HEV in S10-3 cells. As shown in Fig. 6K, HEV-Q421A mutant showed a resistance of GRP75-mediated degradation of ORF2, which is similar to that of overexpressed proteins in HEK-293T cells.

**Fig 7:**
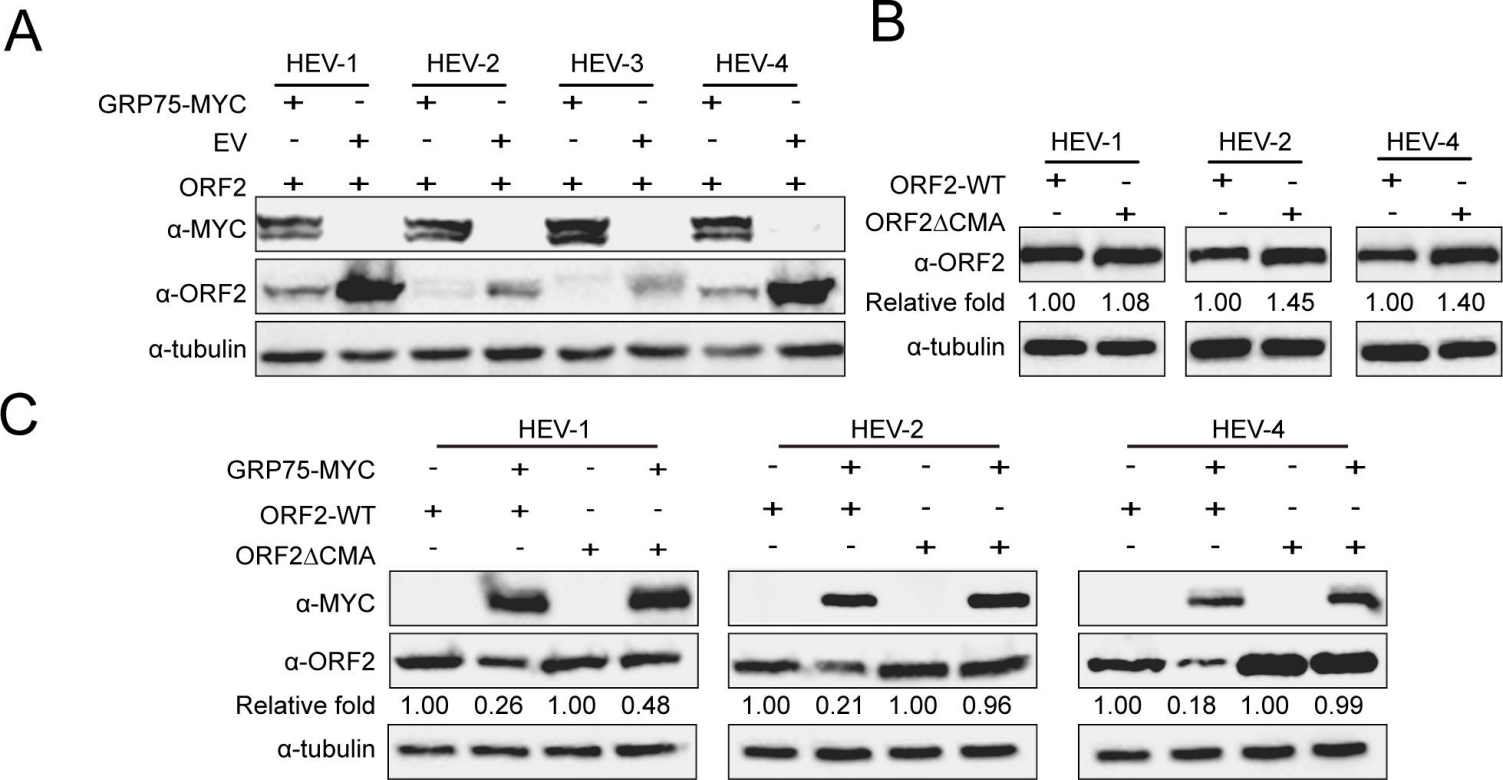
GRP75-mediated degradation of ORF2 through CMA is not HEV genotype-specific. (**A**) GRP75-mediated inhibition of HEV-ORF2 expression is not genotype-specific. HEK-293T cells were transfected with plasmids encoding HEV-ORF2 proteins from HEV-1, HEV-2, HEV-3, and HEV-4, along with a GRP75-encoding plasmid. After 48 h, cells were harvested for western blotting to assess GRP75 and HEV-ORF2 expression levels. GAPDH served as a loading control to normalize total protein input. (**B**) Deletion of KFERQ-like motif in ORF2 of HEV-1, 2, and 4 led to evaluation of ORF2 protein level. HEK-293T cells were co-transfected with wild-type ORF2 (WT ORF2) from HEV1, 2, and 4, or KFERQ-like motif-deleted ORF2 mutant (ORF2ΔCMA) from HEV1, 2, and 4. After 48 h, cells were harvested for western blot analysis to assess ORF2 protein levels. Tubulin served as a loading control. (**C**) Deletion of the all CMA motif in ORF2 of HEV-1, 2, and 4 conferred resistance to GRP75-mediated degradation. HEK-293T cells were co-transfected with a GRP75-MYC-encoding plasmid or empty vector (EV), along with either wild-type ORF2 (WT ORF2) or KFERQ-like motif-deleted ORF2 mutants (ORF2ΔCMA) from HEV1, 2, and 4. After 48 h, cells were harvested for western blot analysis to assess ORF2 protein levels. Tubulin served as a loading control.

Besides HEV-3, since sequence analysis suggested that KFERQ-like motifs of ORF2 were conserved among 4 HEV genotypes, to determine whether GRP75-mediated degradation of HEV-ORF2 is conserved across different HEV genotypes, we synthesized ORF2-encoding sequences from HEV-1, HEV-2, and HEV-4 and further co-transfected them with GRP75 into HEK-293T cells. Similar to HEV-3 KernowC1-p6, GRP75-mediated degradation of ORF2 was observed in all genotypes, indicating that GRP75-mediated degradation of ORF2 is a universal phenomenon and not strain-specific (Fig. 7A). Conversely, ORF2 mutants bearing deletions of all three KFERQ-like motifs were generated for HEV-1, HEV-2, and HEV-4 as well and compared with corresponding wild-type ORF2s. Except for HEV-1, deletion of KFERQ-like motifs in ORF2 of HEV-2 and HEV-4 resulted in an elevated ORF2 protein level (Fig. 7B), whereas deletion of KFERQ-like motifs in HEV-1 ORF2 only led to a slight elevation of ORF2. However, resistance to GRP75-mediated degradation could also be observed in ORF2ΔCMA mutants from other HEV genotypes, which was similar to that of HEV-3 (Fig. 7C).

**Fig 8:**
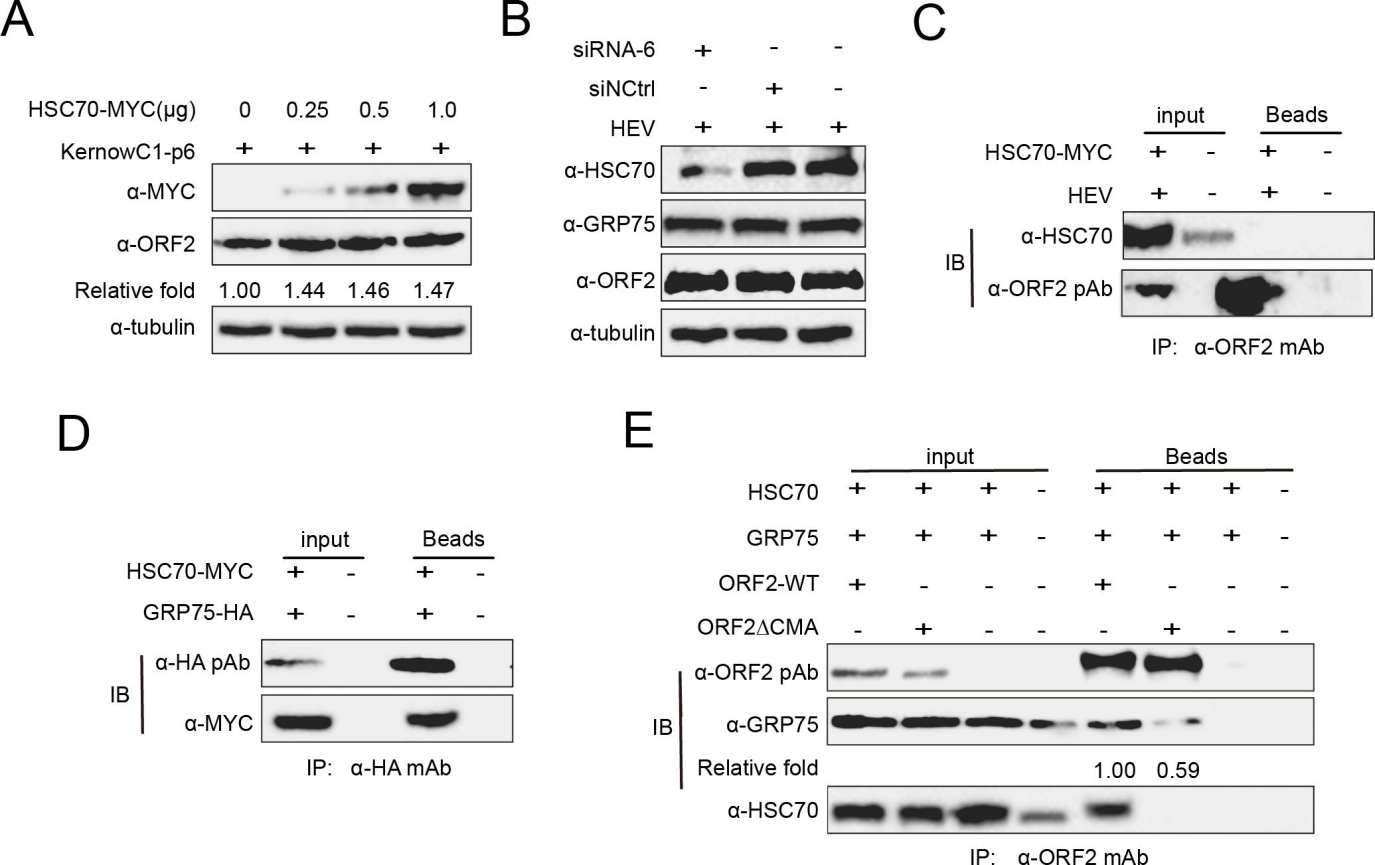
Degradation of ORF2 by GRP75 is dependent on the molecular chaperone HSC70. (**A**) Investigation of HSC70’s role in ORF2 degradation. HEV-replicating S10-3 cells were transfected with increasing doses of a MYC-tagged HSC70 (HSC70-MYC)-encoding plasmid for 48 hours. Cells were then harvested for western blot analysis using MYC-specific monoclonal antibodies (Mab) and ORF2-specific Mab-2G8. Tubulin served as a loading control to normalize protein input. (**B**) Knockdown of HSC70 does not affect HEV-ORF2 expression. HEV-replicating S10-3 cells were transfected with HSC70-specific siRNA (siRNA-6) for 36 h, followed by western blotting using antibodies against HSC70, GRP75, and ORF2. Tubulin served as a loading control. (**C**) Co-immunoprecipitation (Co-IP) assay for ORF2 and HSC70 interaction. HEV-replicating S10-3 cells were transfected with MYC-tagged HSC70 (HSC70-MYC) plasmid for 48 h before being lysed using NP-40 buffer. Co-IP was performed using ORF2-specific Mab-2G8, followed by western blot analysis with HSC70-specific antibody to determine potential interactions. (**D**) Co-IP assay for HSC70 and GRP75 interaction. HEK-293T cells were co-transfected with HA-tagged GRP75 (GRP75-HA) and MYC-tagged HSC70 (HSC70-MYC) plasmids for 48 h before lysis with NP-40 buffer. Immunoprecipitation was carried out using a HA-specific antibody, followed by western blot analysis using MYC-specific antibodies to confirm the interaction. (**E**) Co-IP analysis of the complex formed by HSC70, GRP75, and ORF2. HEK-293T cells were transfected with plasmids encoding HSC70 and GRP75, along with either wild-type HEV-ORF2 (WT ORF2) or an ORF2 mutant lacking the KFERQ motif (ORF2ΔCMA), for 48 h. Cells were then lysed using NP-40 buffer, and immunoprecipitation was performed using ORF2-specific Mab-2G8. Western blotting was conducted using antibodies against HSC70 and GRP75 to detect the corresponding targets in the immunoprecipitated complex.

CMA substrates are known to interact with HSC70 via the KFERQ motif, as HSC70 is the sole molecular chaperone targeting proteins for degradation via the CMA (38). However, when HSC70 was overexpressed in HEV-replicating cells, ORF2 level remained unchanged (Fig. 8A), in contrast to the reduction of ORF2 with GRP75 overexpression. Moreover, HSC70 knockdown using siRNA did not increase ORF2 level (Fig. 8B), and Co-IP analysis in HEV-replicating cells showed that there was no detectable interaction between HSC70 and ORF2 (Fig. 8C). Conversely, during the CMA process, additional chaperone proteins, such as CHIP (carboxyl terminus of HSC70-interacting protein), HSP40, and the HSP70-HSP90 organizing protein (HOP), contribute to substrates targeting to lysosomes (38). These phenomena suggest that GRP75 may function similarly to CHIP or HSP40 in facilitating HSC70-dependent CMA of HEV-ORF2. To assess this possibility, we first analyzed the potential interaction between HSC70 and GRP75, and our results confirmed that HSC70 directly bound to GRP75 when overexpressed in HEK-293T cells (Fig. 8D), implying that HSC70 may require GRP75 as a co-chaperone for CMA degradation of HEV-ORF2. To further investigate whether GRP75 functions as a co-chaperone, we co-transfected HSC70 and GRP75 with wild-type ORF2 or ORF2ΔCMA mutant, then performed Co-IP analysis. As shown in Fig. 8E, in overexpressed cells, both GRP75 and HSC70 could be co-precipitated with wild-type ORF2 (WT ORF2), supporting the notion that ORF2 interacts with HSC70 via GRP75. However, in the ORF2ΔCMA mutant-transfected cell, the interaction between ORF2ΔCMA and GRP75 was significantly weakened and dropped by 50% (Fig. 8E), whereas HSC70 failed to co-precipitate with ORF2ΔCMA. In summary, these results indicate that GRP75 facilitates HEV-ORF2 degradation via the CMA in an HSC70-dependent manner. Moreover, the KFERQ-like motif in ORF2 is essential for its interaction with GRP75 and subsequent complex formation with HSC70 for CMA mediates protein degradation.

### GRP75 antagonizes ORF2’s suppression of IFN production by enhancing the MAVS-TBK1 interaction

**Fig 9:**
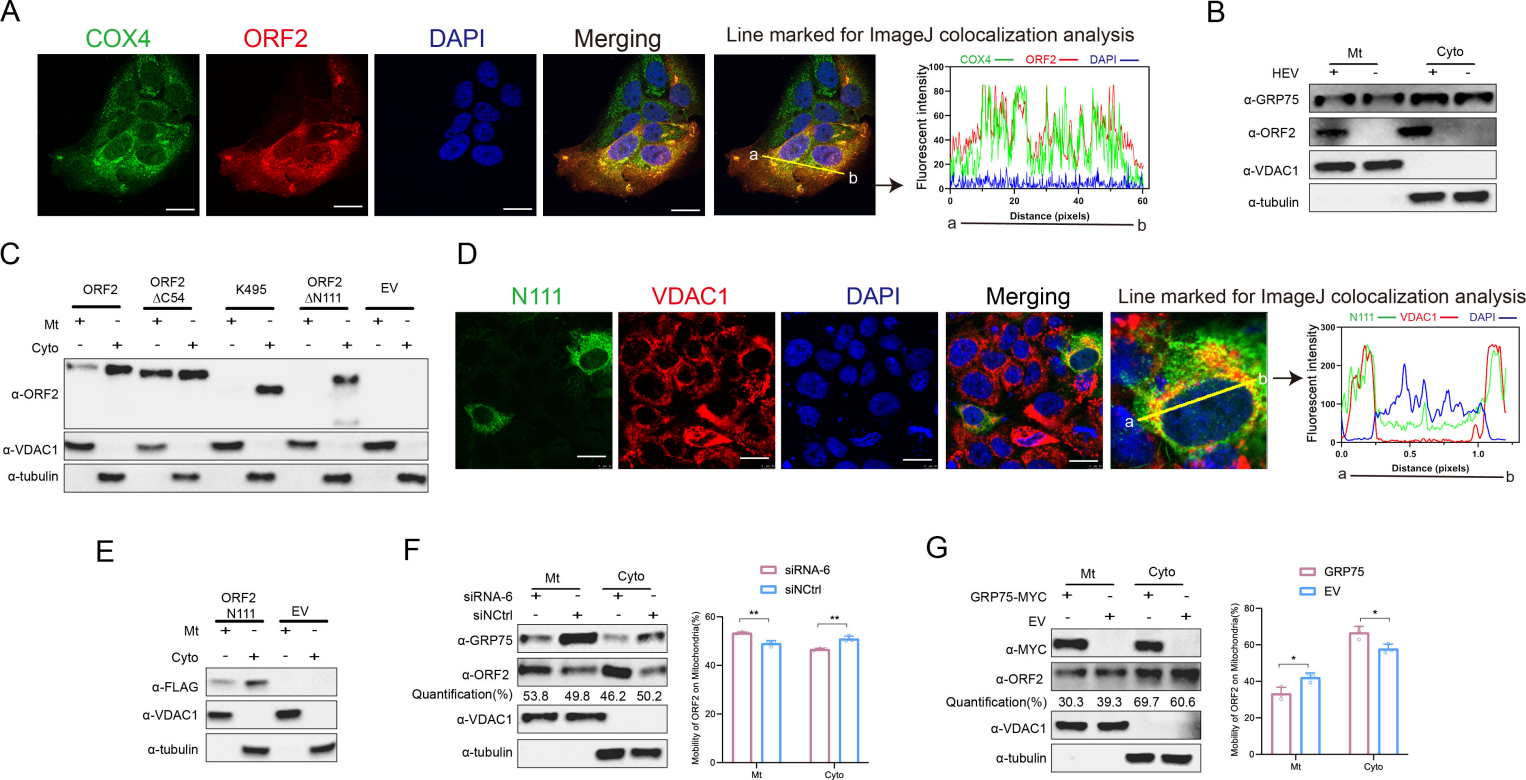
GRP75 blocks the translocation of ORF2 to mitochondria. (**A**) HEV-ORF2 co-localizes with mitochondria. HEV-replicating S10-3 cells were fixed and stained for ORF2 protein (green channel) and mitochondrial protein COX4 (red channel), followed by confocal microscopy. Scale bar = 10 μm. Co-localization across fluorescence channels was analyzed using ImageJ software. (**B**) Mitochondrial fractionation of HEV-replicating S10-3 cells. HEV-replicating S10-3 cells and uninfected S10-3 cells were subjected to mitochondrial extraction using a commercial kit. Extracted fractions were processed with Laemmli sample buffer for SDS-PAGE and western blotting. VDAC1 and tubulin were used as markers to verify successful fractionation of mitochondrial and cytoplasmic proteins, respectively. (**C**) The N-terminal 111 amino acids of ORF2 are responsible for mitochondrial translocation. HEK-293T cells were transfected with plasmids encoding full-length ORF2 (ORF2) or ORF2 truncation mutants, including ORF2ΔC54 (lacking the C-terminal 54 aa), ORF2ΔN111 (lacking the N-terminal 111 aa), p495 (lacking both the N-terminal 111 aa and C-terminal 54 aa), and FLAG-tagged ORF2N111 (N-terminal 111 aa only). After 48 h, cells were subjected to mitochondrial extraction, followed by SDS-PAGE and western blot analysis. VDAC1 and tubulin were used as markers for mitochondrial and cytoplasmic fractions, respectively. (**D**) HEV-ORF2-N111 colocalized with mitochondrial marker. Normal S10-3 cells transfected with plasmids expressed FLAG-tagged ORF2-N111 truncation for 48 h. Next, cells were fixed and stained for FLAG antibody protein (green channel) and mitochondrial protein VDAC1 (red channel), followed by confocal microscopy. Scale bar = 10 μm. Co-localization across fluorescence channels was analyzed using ImageJ software. (**E**) HEV-ORF2- localized in isolated mitochondrial fraction. Normal S10-3 cells transfected with plasmids expressed FLAG-tagged ORF2-N111 truncation for 48 h. Then, the cells were subjected to mitochondrial extraction. Extracted fractions were processed with Laemmli sample buffer for SDS-PAGE and western blotting. VDAC1 and tubulin were used as markers to verify successful fractionation of mitochondrial and cytoplasmic proteins, respectively. (**F**) Knockdown of GRP75 promotes ORF2 translocation to mitochondria. HEV-replicating S10-3 cells were transfected with GRP75-specific siRNA-6 or control siRNA, followed by mitochondrial extraction. Western blotting was performed to assess ORF2 protein levels in mitochondrial and cytoplasmic fractions, with VDAC1 and tubulin used as fractionation markers. Error bars represent quantification from three independent experiments. All data are presented as mean ± SD and were analyzed using Student’s *t*-test. **, *P* < 0.01. (**G**) Overexpression of GRP75 inhibits ORF2 translocation to mitochondria. HEV-replicating S10-3 cells were transfected with a plasmid encoding MYC-tagged GRP75 or empty vector (EV) before mitochondrial extraction. Western blotting was performed to assess ORF2 protein levels in different fractions, with VDAC1 and tubulin serving as markers of successful fractionation of mitochondria and cytoplasm. Error Bar represents quantification from three independent experiments. All data are presented as mean ± SD and were analyzed using Student’s *t*-test. *, *P* < 0.05.

In addition to its role in CMA-mediated degradation of HEV-ORF2, we notice that GRP75 is a mitochondrial protein, also known as mitochondrial heat-shock protein 70 kDa (mtHsp70) (39). To determine whether the interaction between GRP75 and ORF2 affects the subcellular localization of ORF2, we performed confocal microscopy on HEV-replicating cells. Co-localization analysis indicated that a small fraction of ORF2 was presented in mitochondria marked by COX4, whereas the majority remained in the cytoplasm (Fig. 9A). To confirm these findings, we conducted a mitochondria isolation assay, which showed that a portion of ORF2 localized to mitochondria, whereas the most amount displayed a cytoplasmic location (Fig. 9B).

Mitochondria have specialized pathways for protein import and sorting (40), and the presequence pathway is the predominant import route for mitochondrial targeting proteins (41), which is characterized by a cleavable N-terminal mitochondrial-targeting signal (41). Based on the putative functional regions for forming VLPs by recombinant ORF2 proteins expressed in insect cells, we truncated full-length ORF2 into N111, p495, and ORF2ΔC54 truncations. Mitochondrial isolation revealed that deletion of the N111 region completely abolished ORF2’s mitochondrial localization (Fig. 9C). Conversely, when the ORF2-N111 truncation was expressed in cells, confocal microscopy confirmed ORF2-N111 co-localization with the mitochondrial marker VDAC1 (Fig. 9D), suggesting that N111 is responsible for mitochondrial transport. Meanwhile, in mitochondrial isolation assay, a portion of ORF2-N111 was detected in the mitochondrial fraction (Fig. 9E), suggesting that ORF2-N111 alone is sufficient for mitochondrial targeting.

To assess the role of GRP75 on ORF2 mitochondrial localization, we conducted siRNA-mediated knockdown of GRP75 in HEV-replicating cells. It appeared that GRP75 depletion by siRNA led to a moderate increase in HEV-ORF2 accumulation in mitochondria (Fig. 9F). Since GRP75 knockdown increased the ORF2 level, we further quantified the percentage of GRP75 translocating from cytoplasm into mitochondria in GRP75-knockdown cells and compared it with control siRNA-transfected cells. The result still showed a moderate increase in ORF2’s translocation into mitochondria. On the contrary, once plasmid encoding GRP75 was transfected, a moderate reduction of HEV-ORF2’s mitochondrial translocation could be observed compared with empty vector transfected cells when quantification of translocated ORF2 between two groups was conducted (Fig. 9G). Therefore, these results suggest that GRP75 also plays a moderate role in affecting ORF2’s mitochondrial transport.

**Fig 10:**
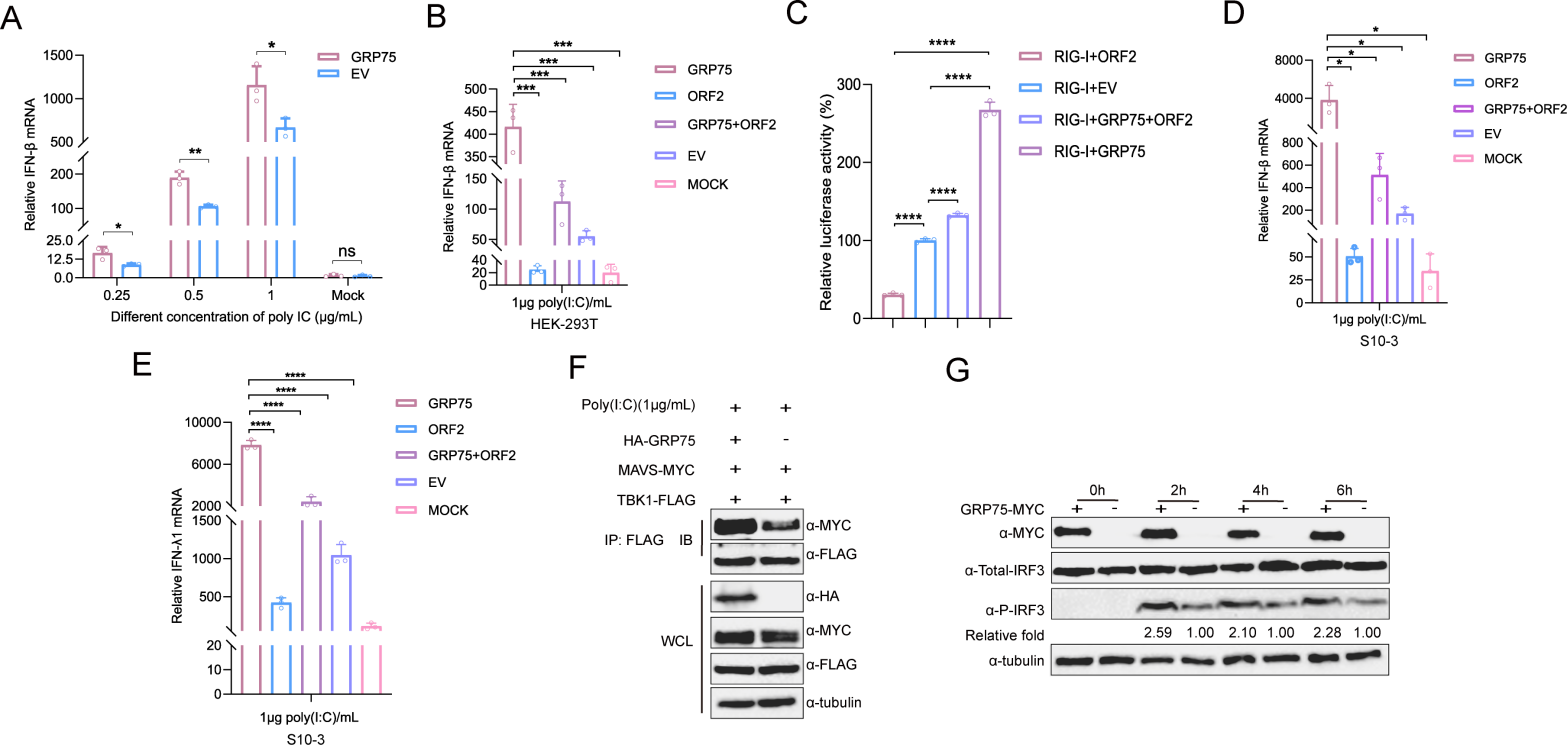
GRP75 antagonizes ORF2’s ability to block IFN induction by enhancing the MAVS-TBK1 interaction. (**A**) GRP75 enhances poly(I:C)-induced IFN-β expression. HEK-293T cells were transfected with a plasmid encoding MYC-tagged GRP75 or empty vector (EV) for 24 h. Cells were then further transfected with different doses of high-molecular-weight (HMW) poly(I:C) for 24 h, whereas a non-transfected group served as a control. After treatment, cells were harvested using TRIzol reagent, and qPCR was performed to measure IFN-β mRNA levels. GAPDH transcription from the same cDNA was used as an internal control. All data are presented as mean ± SD and analyzed using Student’s *t*-test. *, *P* < 0.05; **, *P* < 0.01; ns, not significant. All results are based on at least three independent biological replicates. (**B**) GRP75 antagonizes HEV-ORF2’s inhibition of IFN-β induction in HEK-293T cells. HEK-293T cells were co-transfected with a plasmid encoding MYC-tagged GRP75 or empty vector (EV), along with ORF2 encoding plasmid for 24 h. Then, cells were further transfected with 1 μg HMW poly(I:C) for 24 h to induce expression of IFN-β. Next, cells were harvested using TRIzol reagent, and qPCR was performed to measure IFN-β mRNA levels. GAPDH transcription from the same cDNA was used as an internal control. All data are presented as mean ± SD and analyzed using Student’s *t*-test. ***, *P* < 0.001; ns, not significant. (**C**) GRP75 antagonizes HEV-ORF2’s inhibition of RIG-I activated IFN-β. HEK-293T cells were co-transfected with a plasmid encoding RIG-I N terminal (RIG-I N), MYC-tagged GRP75, and ORF2, along with IFN-β promoter reporter plasmid and pRL-TK control plasmid for 24 h. Then, cells were lyzed for Dual-luciferase reporter assay to evaluate activation of IFN-β promoter. Firefly luciferase in cells transfected with IFN-β promoter reporter and pRL-TK control plasmid was set as 1-fold. All data are presented as mean ± SD and analyzed using Student’s *t*-test for indicated groups. ****, *P* < 0.0001. (**D**) GRP75 antagonizes HEV-ORF2’s inhibition of IFN-β induction in S10-3 cells. S10-3 cells were co-transfected with a plasmid encoding MYC-tagged GRP75 or empty vector (EV), along with ORF2 encoding plasmid for 24 h. Then, cells were further transfected with 1 μg HMW poly(I:C) for 24 h to induce the expression of IFN-β. Next, cells were harvested using TRIzol reagent, and qPCR was performed to measure IFN-β mRNA levels. GAPDH transcription from the same cDNA was used as an internal control. All data are presented as mean ± SD and analyzed using Student’s *t*-test. *, *P* < 0.05. (**E**) GRP75 antagonizes HEV-ORF2’s inhibition of IFN-λ1 induction similar to type I IFN. S10-3 cells were co-transfected with plasmids similar to above, then cells were further transfected 1 μg HMW poly(I:C) for 24 h to induce expression of IFN-λ1. Next, cells were harvested using TRIzol reagent, and qPCR was performed to measure IFN-β mRNA levels. GAPDH transcription from the same cDNA was used as an internal control. All data are presented as mean ± SD and analyzed using Student’s *t*-test for indicated groups. ****, *P* < 0.0001. (**F**) GRP75 enhances the interaction between TBK1 and MAVS. HEK-293T cells were co-transfected with plasmids encoding FLAG-tagged TBK1 and MYC-tagged MAVS, along with either an HA-tagged GRP75 plasmid or EV. After 48 h, cells were transfected with 1 μg poly(I:C) before lysed using NP-40 buffer, and IP was performed using a TBK1-specific antibody. Western blotting was conducted using a MAVS-specific antibody to assess the levels of MAVS-TBK1 complexes pulled down by IP. (**G**) GRP75 enhances downstream IRF3 activation. HEK-293 cells stably expressing Venus-tagged IRF3 (HEK-293-Venus-IRF3) were transfected with a plasmid encoding MYC-tagged GRP75 or empty vector (EV) for 24 h. Cells were then further transfected with 1 μg poly(I:C) for 0, 2, 4, or 6 h. After treatment, cells were harvested using Laemmli sample buffer, and western blot analysis was performed using antibodies specific for phosphorylated IRF3 (pIRF3-S396) and total IRF3. Tubulin served as a loading control.

Previous reports demonstrated that the N-terminal 111 amino acids of HEV-ORF2 inhibit type I IFN production by blocking IRF3 phosphorylation through interactions with MAVS, TBK1, and IRF3 (27). Given the critical role of mitochondria on IFN induction, we hypothesized that GRP75-mediated ORF2 degradation and cytoplasm sequestration counteracts ORF2’s suppression of IFN induction. To test this, we transfected HEK-293T cells with GRP75, then stimulated RIG-I-mediated IFN production using poly(I:C) transfection, and followed by qPCR analysis. The results showed that overexpression of GRP75 significantly enhanced IFN-β transcription compared with empty vector control (Fig. 10A), indicating that GRP75 promotes IFN signaling. Meanwhile, when co-transfected with ORF2 and followed by poly(I:C) stimulation, the presence of GRP75 could mitigate ORF2-mediated suppression of IFN induction (Fig. 10B). Consistent with this observation, in IFN-β reporter assay, presence of GRP75 could enhance RIG-I N-terminal induced activation of IFN-β promoter, or mitigate ORF2-mediated suppression on IFN-β promoter activation (Fig. 10C). Moreover, in HEV-permissive S10-3 cells, similar results could be observed as well (Fig. 10D). Meanwhile, as a previous report suggested that HEV replication in HepG2/C3A cells stimulated production of type III IFNs (42); therefore, we also evaluated IFN-λ1 level, and the result suggested overexpression of GRP75 not only enhanced IFN-λ1 transcription level but also mitigated ORF2-mediated suppression on IFN-λ1 transcription, which is similar to that of type I IFN (Fig. 10E).

Since both GRP75 and MAVS are mitochondrial proteins, we performed Co-IP assays to determine whether GRP75 enhances the MAVS-TBK1 interaction. GRP75 overexpression strengthened the MAVS-TBK1 interaction after poly(I:C) stimulation, suggesting enhanced downstream signaling (Fig. 10F). In agreement with this, we assessed IRF3 phosphorylation in GRP75-overexpressing cells and found that GRP75 significantly increased IRF3 phosphorylation following poly(I:C) stimulation (Fig. 10G), further supporting the role of GRP75 in promoting IFN production.

**Fig 11:**
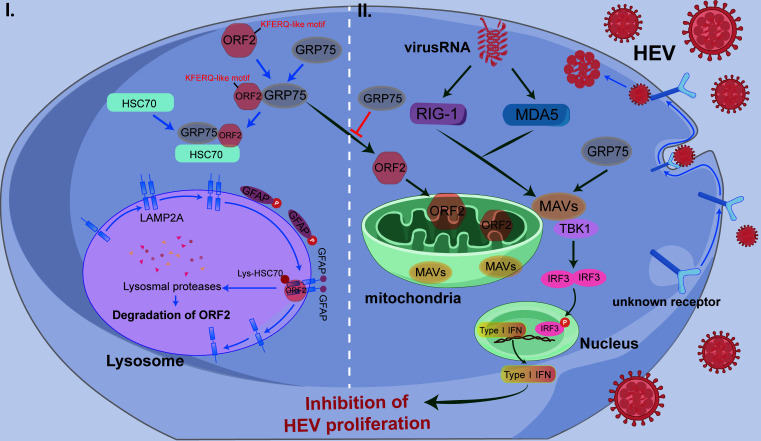
Illustration of GRP75 blocks hepatitis E virus infection through chaperone-mediated autophagy CMA-mediated degradation of HEV-ORF2 and promoting IRF3 activation.

In conclusion, our findings indicate that GRP75 restricts HEV replication through two distinct mechanisms (Fig. 11). First, GRP75 interacts with HEV-ORF2 and promotes its degradation through the CMA pathway. Second, GRP75 inhibits ORF2 mitochondrial transport, reducing its function as an IFN antagonist, while simultaneously enhancing IRF3 activation to promote IFN production. These results establish GRP75 as a novel antiviral factor against HEV infection.

## DISCUSSION

GRP75, a member of the HSP70 chaperone family (39), is also known as heat-shock protein family A member 9 (HSPA9), peptide-binding protein 74 (PBP74), mtHsp70, or mortalin (39). GRP75 plays a crucial role in mitochondrial function and cellular stress responses, making it essential for cellular homeostasis and protection under various physiological conditions (39). Similar to HSP70, GRP75 contains two highly conserved functional domains, NBD and SBD, which are structurally homologous to those found in other HSP70 family members (43). These domains enable GRP75 to function as a molecular chaperone through iterative cycles of substrate interaction, modulated by ATP binding and hydrolysis-driven conformational changes (43). The SBD of GRP75 exhibits strong specificity for hydrophobic amino acid residues, sequestering substrate proteins within the SBDβ subdomain. ATP hydrolysis in the NBD facilitates this process, transitioning GRP75 into an ADP-bound conformation, which reinforces the substrate-SBD interaction and significantly increases substrate binding affinity (43).

Beyond its physiological function, GRP75 has also been implicated in viral infections, particularly due to its role in macromolecule endocytosis, a process crucial for viral entry into host cells (44). Conversely, existing studies suggest that GRP75 acts as a restriction factor for certain RNA viruses. For example, GRP75 inhibits the replication of porcine epidemic diarrhea virus (PEDV) by downregulating clathrin expression, thereby impairing the endocytic process (45). Additionally, Shelton et al. demonstrated that GRP75 interacted with Nef, an early HIV protein, to inhibit the cellular release of HIV virions (46). Regarding HEV, previous reports have shown that the HEV-ORF2 protein interacts with HSP72 (HSPA2) and GRP78 (HSPA5), two other members of the HSP70 chaperone family (47, 48). However, the biological function of these interactions remains unclear. In this study, we identified GRP75 as a novel interacting partner of the HEV capsid protein ORF2 through its substrate-binding domain, further supporting the role of HSP70 family members in HEV replication. Knockdown of GRP75 in HEV-replicating cells enhanced HEV infection, whereas GRP75 overexpression significantly suppressed HEV infection. These findings suggest that GRP75 functions as a restriction factor for HEV infection, consistent with its inhibitory role played during PEDV and HIV infections. Notably, our results revealed that GRP75 inhibited HEV infection by binding to ORF2 via its SBD domain and further facilitating ORF2 degradation through the CMA pathway.

Autophagy consists of three primary forms: macroautophagy, microautophagy, and CMA (32, 33). Although macroautophagy and microautophagy can be either selective or non-selective, CMA is distinguished by its strict substrate specificity, ensuring the targeted degradation of specific proteins (49). Proteins targeted for CMA-dependent degradation must contain a KFERQ sequence or a KFERQ-like motif. Our study indicates that GRP75 mediates HEV-ORF2 degradation via the lysosomal pathway. Sequence analysis of ORF2 from four HEV genotypes known to infect humans revealed three potential KFERQ-like motifs, suggesting their importance for CMA recognition. Consistent with this hypothesis, deletion of these KFERQ-like motifs significantly increased HEV-ORF2 protein level and conferred ORF2 protein resistance to GRP75-mediated degradation, confirming that ORF2 degradation by GRP75 is KFERQ-like motif-dependent. Moreover, a specific interaction between ORF2 and LAMP2A was detected in HEV-replicating cells as well, further supporting that GRP75-mediated ORF2 degradation is CMA-dependent.

To date, HSC70 (HSPA8) is the only known chaperone that mediates CMA by interacting with KFERQ motifs in CMA substrates (50–52). Other co-chaperones, including CHIP, HSP40, and HOP, contribute to CMA by facilitating substrate targeting to lysosomes but function in an HSC70-dependent manner (53). Our data suggest that GRP75 is involved in CMA-dependent degradation of HEV-ORF2. Since both GRP75 and HSC70 are constitutively expressed and belong to the same molecular chaperone family, GRP75 may act as a co-chaperone in the CMA process, similar to that of CHIP or HSP40. To explore this possibility, we further examined the interaction between HSC70 and GRP75 and confirmed that GRP75-mediated ORF2 degradation aligns with the canonical CMA pathway. Although HSC70 was not detected to play a direct role on ORF2 degradation in HEV-replicating cells, it was able to form a complex with GRP75 and ORF2 when overexpressed in HEK-293T cells, whereas deletion of the KFERQ-like motifs in HEV-ORF2 impaired this complex formation. Interestingly, sequence analysis reveals that the KFERQ-like motifs in HEV-ORF2 are atypical compared with classical KFERQ motifs, implying that GRP75 may interact with these non-canonical CMA motifs presented in viral proteins, thereby facilitating their degradation through CMA during viral replication. Therefore, further investigation is required to understand if GRP75 could recognize more non-canonical CMA motifs from other viruses.

During a viral infection, a continuous evolutionary arms race occurs between the virus and host, wherein both entities adapt to gain a selective advantage (54). The host activates innate immune responses, particularly the IFN system, to eliminate the virus or restrict its replication. For instance, cytoplasmic viral RNA is recognized by RIG-I-like receptors (RLRs) (55), which trigger MAVS aggregation on mitochondria, leading to antiviral signaling (56). MAVS subsequently recruits E3 ubiquitin ligases, such as tumor necrosis factor receptor-associated factors 2, 3, and 6 (TRAF2, TRAF3, and TRAF6) (57), which activate TBK1, a serine/threonine kinase. Activated TBK1 phosphorylates IRF3, allowing its dimerization and nuclear translocation, where it drives IFN production and the expression of IFN-stimulated genes (ISGs) (57). Conversely, viruses evolve immune evasion strategies to persist and propagate. Previous studies indicated that HEV-ORF2 antagonized RIG-I-mediated IFNs production by inhibiting IRF3 activation through interaction with MAVS, TBK1, and IRF3 (27, 58, 59), whereas deletion of the N111 region from full-length ORF2 abolished the role of ORF2 in antagonizing RIG-I-dependent IFN response (58). Given that the N111 region of HEV-ORF2 contains a hydrophobic domain and is cleaved from ORF2-p495, our results confirmed that ORF2-N111 contained mitochondria-targeting sequence to mediate the mitochondrial translocation of ORF2, where it likely allows ORF2 to interfere with IFN induction (40). However, this process could be blocked by GRP75. GRP75 knockdown enhanced ORF2’s translocation to mitochondria, whereas GRP75 overexpression reduced it, suggesting that GRP75 counteracts ORF2-N111’s inhibition of MAVS-mediated TBK1 activation. Although our data indicated GRP75 overexpression did not lead to HEV-RNA replication inhibited in the HEV-replicon system, it might be caused by the established HEV-RNA replication in cells and IFN antagonist function of HEV-ORF1 protein expressed by the HEV-replicon (60–62). Such a replicon system cannot fully simulate HEV replication and viral-host interaction processes *in vivo*. It is possible that GRP75-mediated IFN enhancement plays more role in blocking HEV from infection of fresh cells rather than slowing down viral replication speed in HEV-infected cells. Therefore, we still propose that GRP75 contributes to IFN pathway activation, reinforcing host antiviral defenses against HEV. Our results indicate that GRP75 enhances the MAVS-TBK1 interaction, promoting IFN-β production and antiviral gene expression.

In conclusion, our study demonstrates that GRP75 functions as an inhibitor of HEV replication through its specific interaction with HEV-ORF2. GRP75 restricts HEV replication via two principal mechanisms: (i) targeting ORF2 for HSC70-dependent CMA degradation and (ii) enhancing IFN production by counteracting ORF2’s antagonistic effects on IFN signaling and promoting IRF3 activation, thereby establishing an antiviral state to suppress HEV replication.

## MATERIALS AND METHODS

### Cells, viruses, and chemicals

S10-3 cells, a subclone of Huh-7 hepatoma cell line (18), HepG2/C3A cell, normal HEK-293, and HEK-293T cells were maintained in Dulbecco’s modified Eagle medium (DMEM; Thermo Fisher Scientific, Waltham, MA, United States) supplemented with 10% FBS (Thermo Fisher Scientific). The method for developing HEK-293 cell with stable expression of VenusC1-tagged IRF3 (HEK293-IRF3) was previously described (60). Briefly, HEK293-IRF3 cells were established by transfection of the cells with VenusC1-IRF3 and selection under the presence of G418 (Sigma-Aldrich, St. Louis, MO, USA) in cell culture medium at the concentration of 500 μg/mL. The surviving cells after 2 weeks of G418 treatment were cloned by limited dilution and cell sorting by flow cytometry. The HepaRG cells were previously purchased from commercial suppliers and stored in Harbin Veterinary Research Institute. The HepaRG cells were maintained in RPMI 1640 medium (Thermo Fisher Scientific) supplemented with 10% FBS (Thermo Fisher Scientific).

The primary porcine hepatocytes were freshly isolated from newly born piglets purchased from a PRRSV-free pig farm near Yangling, Shaanxi, and further screened for swine HEV, CSFV, PCV2, and ASFV along with corresponding antibodies. Only piglets negative for all examined pathogens and antibodies against HEV, PRRSV, and ASFV were selected for primary porcine hepatocytes isolation. Briefly, after euthanization of piglets and dissection of abdominal cavity, the inferior vena cava was ligatured, followed by liver perfusion using pre-heated (37°C) D-Hanks Balanced Salt Solution (D-HBSS) supplemented with 0.5 mM EDTA through hepatic portal vein to remove the blood from liver. Next, plain D-HBSS was perfused into the liver again to remove EDTA before D-HBSS containing 0.1% collagenase IV (Thermo Fisher Scientific) and 5 mM Ca^2+^ was perfused into the liver for tissue digestion. After softening of the hepatic lobule, cell suspension containing primary porcine hepatocytes was harvested by the incision of Glisson’s capsule and washing the liver using plain William’s E Medium (Thermo Fisher Scientific). After passing the single-cell filter, the porcine hepatocyte suspension was further purified using 50% and 25% Percoll (Merck, Darmstadt, Germany) for density gradient centrifugation before further experiment. Purified primary porcine hepatocytes were maintained using William’s E Medium containing 10% FBS, 0.5 μg/mL insulin (BasalMedia, Shanghai, China), 100nM Dexamethasone, and 20 ng/mL human epidermal growth factor (Sino Biological, Beijing, China).

Full-length RNA of HEV-3 KernowC1-p6 strain (GenBank Accession: M74506.1) and KernowC1-p6-zsGreen replicon were obtained by *in vitro* transcription from plasmids of pSK-HEV-kernowC1-p6 and pSK-HEV-kernowC1-p6-zsGreen digested with *MluI* (63, 64), using AmpliCap-Max T7 High Yield Message Maker Kit (Cellscript, Madison, WI, USA). The HEV-3 KernowC1-p6 infectious clone bearing ORF2-Q421A mutation was generated by site-directed mutagenesis in pSK-HEV-kernowC1-p6 plasmid for *in vitro* transcription in the same manner. The capped RNA yielded from i*n vitro* transcription was further purified using TRIzol Reagent (Thermo Fisher Scientific) according to the manufacturer’s protocol.

Evaluation of assembled HEV particles was conducted by infecting fresh HepG2/C3A cells using cell culture supernatant collected from cells transfected with HEV-RNA. Briefly, the cell culture supernatant was concentrated for 20-fold using 100 kDa ultrafiltration tube (EMD Millipore, Burlington, MA, USA) and then added to fresh HepG2C3A cells and incubated for 6 days while a half-replacement of cell culture medium was conducted at day 3 post-inoculation. After 6 days of incubation, the infected cells were fixed and stained using HEV-ORF2 antibody for observation under immunofluorescence microscopy.

Transfection of DNA plasmids into different mammalian cells was conducted using FuGENE HD (Promega, Madison, WI, USA) according to the manufacturer’s instructions. Transfection of HEV-RNA was conducted using an optimized protocol with DMRIE-C (Thermo Fisher Scientific). Briefly, after cells reached 70% confluence in a 12-well plate, the cell culture medium was discarded, and the monolayer cells were washed with PBS twice, followed by the addition of 0.5 mL serum-free Opti-MEM (Thermo Fisher Scientific) to each well. For each well, 1 µg RNA was added into 50 µL Opti-MEM and mixed evenly with 4 µL DMRIE-C. After incubation at room temperature (RT) for 20 min, the mixture was added to cells, and 1 mL DMEM with 10% FBS or other complete mediums (William’s E Medium, RPMI 1640) was added to each well at 5 h post-transfection. HEV-RNA transfected cells were cultured at 37°C for 7 days. HEV-3 KernowC1-p6 virus was rescued from the transfected cell culture supernatant and concentrated using a previously described protocol (60), and the virus was used to inoculate HepG2/C3A cells for generating the cells stably infected with HEV. Other chemicals used in this study, such as MG132, chloroquine (CQ), 3-Methyladenine (3-MA), and Bafilomycin A1 (Baf-A1) were obtained from Sigma-Aldrich. poly(I:C) (HMW, Invivogen, San Diego, CA, USA), a synthetic analog of double-stranded RNA (dsRNA), was used to induce interferon production. The cells were transfected with the poly(I:C) at a concentration of 1 µg/mL for stimulation of type I interferon induction and further analysis.

### Plasmids and antibodies

The ORF2 cDNA sequence of HEV-3 KernowC1-p6 strain was amplified from infectious clone pSK-HEV-KernowC1-p6, followed by ligation to pCAGEN plasmids to fuse with a C-terminal MYC tag or without any tag. The ORF2 cDNA sequence of HEV-1 Sar55 strain was amplified from infectious clone pSK-HEV-2 (GenBank Accession: AF444002). The ORF2 cDNA sequences from HEV-2 (GenBank Accession: M74506) and HEV-4 (GenBank Accession: KF176351) were artificially synthesized by Genscript Co. Ltd (Nanjing, China). The ORF2 mutants from all four HEV genotypes bearing deletion of three KFERQ-like motifs were artificially synthesized for Genscript Co. Ltd as well. The rest ORF2 mutant bearing deletion of the second KFERQ-like motif or single point mutation was generated by site-directed mutagenesis on wild-type ORF2 plasmid. The cDNA sequences of human and porcine GRP75 were cloned from HEK-293T and immortalized porcine alveolar macrophage CRL2843, followed by ligation to pCAGEN plasmids to fuse with a C-terminal MYC tag. The ATPase enzymatic inactivation mutant of GRP75 (GRP75^K73M^) was generated by mutagenesis on wild-type human GRP75 plasmid. Truncations of human GRP75 were generated by subcloning using full-length GRP75 plasmid as template. Primers for these plasmid constructions and corresponding DNA sequences were listed in Table 1.

**TABLE 1:** Primers and corresponding sequences

Primer	Sequence (5'−3')	Description
JVHEV-F	GGTGGTTTCTGGGGTGAC	Primer pairs for Taqman probe
JVHEV-R	AGGGGTTGGTTGGATGAA	
JVHEVP	TGATTCTCAGCCCTTCGC	Taqman Probe
ORF1-F	AATCCAGACCACGAGCCG	qPCR for detection of HEV(+)/(-) RNA
ORF1-R	ATCGCACCAGGGTTAGCG	
KORF2-F	CATCATTTTGGCAAAGAATTCCTCGAGATGTGCCCTAGGGTTGTTCTG	Cloning of ORF2 to pCAGEN vector
KORF2-R	GCAGCCTGCACCTGAGGAGTGCGGCCGCAGACTCCCGGGTTTTGCCTA	
K239-F	ACAAGCTTGCGGGGCCGCCAGAATTCATCGCCCTGACACTGTTCAA	Cloning of K239 to pET21b vector
K239-R	CTCTAGAGTCGACTGGTACCGATATCGCGGCCGAGTGTGGGGCTAATA	
LAMP2A-F	AGATTATGCTGAATTCCGTCTCCTCGAGATGGTGTGCTTCCGCCTCTT	Cloning of LAMP2 to pCAGEN vector
LAMP2A-R	GAGATGAGTTTCTGCTCCGCGGCCGCAAATTGCTCATATCCAGCAT	
GRP75-F	ATTATGCTGAATTCCGTCTCCTCGAGATGATAAGTGCCAGCCGAGC	Cloning of GRP75 to pCAGEN vector
GRP75-R	CAGAGATGAGTTTCTGCTCCGCGGCCGCCTGTTTTTCCTCCTTTTGAT	
GRP75-F	CCGACGACGACGACAAGGGGCCATGGATGATAAGTGCCAGCCGAGC	Cloning of GRP75 to pET30b vector
GRP75-R	CAGTGGTGGTGGTGGTGGTGCTCGAGCTGTTTTTCCTCCTTTTGAT	
NBD-F	TCCAGATTATGCTGAGCAGAAACTCATCTCTGAAGAGGATCTGGAATTCCGT	Cloning of NBD to pCAGEN vector
NBD-R	TCAGAGATGAGTTTCTGCTCCGCGGCCGCAATCACTTCTCCTATGTCA	
SBD-F	TTCCAGATTATGCTGAGCAGAAACTCATCTCTGAAGAGGATCTGGAATTCCGT	Cloning of SBD to pCAGEN vector
SBD-R	CAGAGATGAGTTTCTGCTCCGCGGCCGCCTGTTTTTCCTCCTTTTGAT	
GRP75-qF	CTTGGGGCACACAGCAAAAAATGC	Primer pairs for Taqman probe
GRP75-qF	ACCCGAAGCACATTCAGTCC	
GAPDH-qF	ACAAGGCTGGGGCTCATTTG	
GAPDH-qR	AGGGGCCATCCACAGTCTTC	
Sar55-ORF2-F	AGATTATGCTGAATTCCGTCTCCTCGAGATGCGCCCTCGGCCTATT	Cloning of ORF2 to pCAGEN vector
Sar55-ORF2-R	CAGAGATGAGTTTCTGCTCCGCGGCCGCTAACTCCCGAGTTTTAC	
MEX-ORF2-F	AGATTATGCTGAATTCCGTCTCCTCGAGATGCGCCCTAGGCCTCT	
MEX-ORF2-R	AGAGATGAGTTTCTGCTCCGCGGCCGCCAACTCCCGAGTTTTAC	
sHEV-ORF2-F	ATGATGTTCCAGATTATGCTGAATTCATGCGCTCTCGGGCTCTTCT	
sHEV-ORF2-R	AGTTTCTGCTCCGCGGCCGCGATATCATACTCCCGGGTTTTACCTA	
CMA-F	ATTATGCTGAATTCCGTCTCCTCGAGATGTGCCCTAGGGTTGTTCT	Cloning of ORF2-∆CMA to pCAGEN vector
dCMA1-R	GGGGAGGGCGAAGGGGTCACCCCAGAAACC	
dCMA2-F	GGTTTCTGGGGTGACCCCTTCGCCCTCCCC	
dCMA2-R	ATCGTGTGGGATGGTCTGCGCATTCTCCAC	
dCMA3-F	GTGGAGAATGCGCAGACCATCCCACACGAT	
dCMA3-R	CCGGGTTTTGCCTACAAGCTCAGCAATAGT	
CMA-R	GAGATGAGTTTCTGCTCCGCGGCCGCAGACTCCCGGGTTTTGCCTA	
KORF2-F	CGCCAGAATTCCCGCGGTGGGCAGATCTATGTGCCCTAGGGTTGT	Cloning of ORF2 truncated eukaryotic to pCAGEN vector
KORF2-R	CTCTAGAGTCGACTGGTACCGATATCTTAAGACTCCCGGGTTTTGC	
N111+K495 F	ACGACAAGCTTGCGGCCGCCAGGGAATTCATGTGCCCTAGGGTT	
N111+K495 R	CTCTAGAGTCGACTGGTACCGATATCGCGGCCGAGTGTGGGGCTA	
K495-F	ACAAGCTTGCGGGGCCGCCAGAATTCGCTGTATCACCAGCCCCTG	
K495-R	GGATCCTCTGAGTCGACTGGTACCGATATCGGCCGAGTGTGGGGC	
K495+C54 F	ACAAGCTTGCGGGGCCGCCAGAATTCGCTGTATCACCAGCCCCTG	
K495+C54 R	CTCTAGAGTCGACTGGTACCGATATCTTAAGACTCCCGGGTTTTGC	
N111-F	CGACAAGCTTGCGGCCGCCAGGGAATTCATGTGCCCTAGGGTTGT	
N111-R	TCCTCTAGAGTCGACTGGTACCGATATCAGTCAACGGCGCAGCCC	
KORF2-F	CGCCAGAATTCCCGCGGTGGGCAGATCTATGTGCCCTAGGGTTGT	
KORF2-R	CTCTAGAGTCGACTGGTACCGATATCTTAAGACTCCCGGGTTTTGC	
N111+K495 F	ACGACAAGCTTGCGGCCGCCAGGGAATTCATGTGCCCTAGGGTT	
N111+K495 R	CTCTAGAGTCGACTGGTACCGATATCGCGGCCGAGTGTGGGGCTA	
K495-F	ACAAGCTTGCGGGGCCGCCAGAATTCGCTGTATCACCAGCCCCTG	
K495-R	GGATCCTCTGAGTCGACTGGTACCGATATCGGCCGAGTGTGGGGC	
K495+C54 F	ACAAGCTTGCGGGGCCGCCAGAATTCGCTGTATCACCAGCCCCTG	
K495+C54 R	CTCTAGAGTCGACTGGTACCGATATCTTAAGACTCCCGGGTTTTGC	
IFN-β qF	GATTCATCTAGCACTGGCTGG	qPCR for detection of IFN
IFN-β qR	CTTCAGGTAATGCAGAATCC	
K-1∆CMA-F1	gtggtttctggggtgaccccttcgccctcccctat	Cloning of ORF2∆CMA to pCAGEN vector
K-1∆CMA-R1	gggagggcgaaggggtcaccccagaaac	
K-1∆CMA-R	cacctgaggagtgcggccgcttaagactcccgggttttgc	
K-1∆CMA-F	ttggcaaagaattcctcgagatgtgccctagggttgttct	
K-2∆CMA-F1	ctgtggagaatgcgcagaccatcccacacgatatagat	
K-2∆CMA-R1	tatatcgtgtgggatggtctgcgcattctccacagatgt	
K-3∆CMA-R2	ttaagactcccgggttttgcctacaagctcagcaatagtgg	
K-3∆CMA-R1	actggaatgcacaaccct	
Sar55-ORF2∆CMA-F1	ggcggtggtttctggggtgaccccttcgcaatcccctata	Cloning of ORF2∆CMA to pCAGEN vector
Sar55-ORF2∆CMA-R1	atataggggattgcgaaggggtcaccccagaaaccaccgcc	
Sar55-ORF2∆CMA-F2	acatctgtggagaatgctcaggcaatcccgcatgacatc	
Sar55-ORF2∆CMA-R2	ggtcgatgtcatgcgggattgcctgagcattctccacagatgt	
Sar55-ORF2∆CMA-R3	ctataactcccgagttttacccacaagctcagcgacagtagactg	
Sar55-ORF2∆CMA-R4	gtagactgaaaagcacagccc	
MEX-ORF2∆CMA-F1	gcggtggtttctggggtgaccccttcgcaatcccctatattc	Cloning of ORF2∆CMA to pCAGEN vector
MEX-ORF2∆CMA-R1	gaatataggggattgcgaaggggtcaccccagaaaccaccgc	
MEX-ORF2∆CMA-F2	catcagtggagaatgctcaggctatcccccacgatatcga	
MEX-ORF2∆CMA-R2	tcgatatcgtgggggatagcctgagcattctccactgatg	
MEX-ORF2∆CMA-R3	ctacaactcccgagttttacccacgagctcagcgacagttgactg	
MEX-ORF2∆CMA-R4	gagctcagcgacagttgactggaa	
sHEV-ORF2∆CMA-F1	ggtggtttctggggtgaccccttcgccctcccctat	Cloning of ORF2∆CMA to pCAGEN vector
sHEV-ORF2∆CMA-R1	tataggggagggcgaaggggtcaccccagaaaccacc	
sHEV-ORF2∆CMA-F2	tcagtcgagaacgctcaggctatcccacatgatattg	
sHEV-ORF2∆CMA-R2	gtcaatatcatgtgggatagcctgagcgttctcgactg	
sHEV-ORF2∆CMA-F3	tcaatactcccgggttttacctaccagctcagcaacagtagattg	
sHEV-ORF2∆CMA-R3	agctcagcaacagtagattgaaaggcacaaccc	
sHEV-ORF2∆CMA-R4	ctgcacctgaggagtgcggccgctcaatactcccgggttttac	
K-ORF2^Q421A^-F	catctgtggagaatgcgcaggccgacaagggcattaccatccc	Cloning of ORF2 single-point mutant to pCAGEN vector
K-ORF2^QG424A^-R	gggatggtaatgcccttgtcggcctgcgcattctccacagatg	
	tgtggagaatgcgcagcaagacaaggccattaccatcccacacgatatagattt	
K-ORF2^G424A^-R	aaatctatatcgtgtgggatggtaatggccttgtcttgctgcgcattctccaca	
K-ORF2^GD422A^-F	tctgtggagaatgcgcagcaagccaagggcattaccatcccacacgat	
K-ORF2^D422A^-R	atcgtgtgggatggtaatgcccttggcttgctgcgcattctccacaga	
K-ORF2^K423A^-F	tgtggagaatgcgcagcaagacgccggcattaccatcccacacgatatag	
K-ORF2^K423A^-R	ctatatcgtgtgggatggtaatgccggcgtcttgctgcgcattctccaca	
K-ORF2^I425A^-F	gaatgcgcagcaagacaagggcgccaccatcccacacgatatagatttggg	
K-ORF2^I425A^-R	cccaaatctatatcgtgtgggatggtggcgcccttgtcttgctgcgcattc	

Development of VenusC1-tagged IRF3 plasmid (VenusC1-IRF3) was previously described (60). Development of HEV-ORF2-specific mouse monoclonal antibody (Mab) was previously described (65). Recombinant HEV-ORF2-239 protein of HEV-KernowC1-p6 strain (K239) was prepared as previously described for rabbit polyclonal antibody production (28). The immunization of rabbits and affinity purification of p239-specific polyclonal antibody from immunized rabbits were conducted by Genscript Co. Ltd. The MYC tag Mab (ProteinTech, Wuhan, Hubei, China), anti-GRP75 Mab (Santa Cruz Biotechnology, Santa Cruz, CA, USA), the anti-VDAC1 Mab (ProteinTech), anti-COX4 Mab (ProteinTech), anti-HSC70 Mab (Santa Cruz Biotechnology), anti-LAMP2A polyclonal antibodies (pAb, Thermo Fisher Scientific, 51-2200) and anti-TBK1 Mab (Santa Cruz Biotechnology), anti-MAVS rabbit polyclonal antibodies pAb, (Cell Signaling Technology, Danvers, MA, USA), anti-GRP75 rabbit pAb (ProteinTech), and anti-Phos-IRF3-S396 rabbit Mab (Cell Signaling Technology) were obtained from commercial suppliers. The anti-HEV-ORF1 monoclonal antibody (X domain of Sar55 strain) was previously described (65).

### Pull-down assay and silver staining

To identify the interacting partner of HEV-ORF2, CNBr-activated Sepharose 4B resin (GE Healthcare, Chicago, IL, USA) was washed with a cold solution of 1 mM HCl and conjugated to HEV-3 KernowC1-p6 ORF2-239 protein (K239) following the manufacturer’s instructions at a ratio of 1 mL Sepharose 4B to 4 mg K239 protein. Subsequently, the plasma membrane extract was mixed with K239-conjugated 4B resin and incubated at 4°C for 6 h. Then, the interaction complexes from HEV-ORF2-239-conjugated 4B resin were eluted and subjected to SDS-PAGE, followed by silver staining using a silver stain kit (Solarbio, Beijing, China). Plain Sepharose 4B resin and recombinant SUMO-conjugated Sepharose 4B resin were served as blank control and irrelevant control, respectively.

### RNA interference

S10-3 cells were seeded in 12-well plates at a density of 1 × 10^5^ cells/mL in DMEM containing 10% FBS and cultured overnight. After the cell confluence reached 50%–60%, transfection of the indicated siRNAs was carried out using Lipofectamine RNAiMAX reagent (Thermo Fisher Scientific) in accordance with the manufacturer’s protocol. The siRNA transfection of primary porcine hepatocytes and HepaRG cells was conducted using Lipofectamine RNAiMAX reagent as well. The siRNAs were synthesized by Genepharma Biotech (Shanghai, China). The siRNAs and corresponding sequence used in this study were listed in Table 2.

**TABLE 2:** Sequences for siRNA

Primer	Sequence (5'−3')	Description
si-GRP75-4-F	GGAUUGUCACUGAUCUAAU	siRNA sequence targeting GRP75
siGRP75-4-R	AUUAGAUCAGUGACAAUCC	
si-GRP75-5-F	CGUGAGCAGCAGAUUGUAA	
siGRP75-5-R	UUACAAUCUGCUGCUCACG	
si-GRP75-6-F	GCAGAGACAGGCCACTAAA	
siGRP75-6-R	UUUAGUGGCCUGUCUCUGC	siRNA sequence targeting HSC70
si-HSC70-1-F	CCAAGACUUCUUCAAUGGAAATT	
siHSC70-1-R	UUUCCAUUGAAGAAGUCUUGGTT	
si-HSC70-2-F	UUUCCAUUGAAGAAGUCUUGGTT	
siHSC70-2-R	UUUCCAUUGAAGAAGUCUUGGTT	
si-HSC70-3-F	UUUCCAUUGAAGAAGUCUUGGTT	
si-HSC70—R si-sGRP75-F si-sGRP75-R	UUUCCAUUGAAGAAGUCUUGGTT UCAGAGACAGGCCACUAAGTT AGUCUCUGUCCGGUGATTCTT	siRNA sequence targeting swine GRP75

### Western blotting (WB) analysis

Cells and other samples were harvested by Laemmli sample buffer (Bio-Rad Laboratories, Hercules, CA, USA) containing 2% sodium dodecyl sulfate (SDS) and separated by SDS-polyacrylamide gel electrophoresis (SDS-PAGE). Separated proteins were transferred onto PVDF membranes, probed with antibodies against different targets followed by HRP-conjugated anti-mouse (Thermo Fisher Scientific) or anti-rabbit IgG secondary antibodies (Thermo Fisher Scientific), and then, the protein bands were visualized using enhanced chemiluminescent (ECL) substrate (Bio-Rad Laboratories, GAPDH [ProteinTech], or tubulin [ProteinTech]) was probed using corresponding antibodies from the same membrane as the protein loading control. The mitochondria isolation was conducted using a Mitochondria Isolation kit (Thermo Fisher Scientific) and VDAC1 and tubulin antibodies were used to examine the successful isolation of mitochondrial/cytoplasm fractions in immunoblotting. Chemiluminescence signal acquisition was conducted using a ChemiDoc MP Imaging System (Bio-Rad Laboratories). All western blot images presented in this study represented typical images from at least three independent experiments, and the western blot data quantification was performed using Image Lab software (Version 5.1, Bio-Rad Laboratories). To determine HEV-ORF2 half-life, cycloheximide (Sigma-Aldrich) was used at a final concentration of 100 µg/mL to inhibit universal protein translation. Cell lysate samples were harvested at indicated time points after cycloheximide treatment.

For Far-western blotting analysis, the recombinant GRP75 was used to incubate the PVDF membrane before GRP75 antibodies were added. For recombinant expression of GRP75, the cDNA encoding GRP75 was cloned from cDNA of S10-3 cells, then ligated to pET30a vector. Following transformation into *E. coli* strain BL21 (DE3), cells containing the recombinant construct were cultured in Luria broth (LB) at 37°C then expression of the recombinant protein was induced by culturing the cells in LB containing 0.5 mM isopropyl β-D-thiogalactoside (IPTG, Sigma-Aldrich) at 37°C. Next, the cells were harvested via centrifugation, resuspended in phosphate-buffered saline (PBS), and sonicated. Inclusion bodies containing the recombinant protein were washed with PBS and solubilized in 8 M urea (Sigma-Aldrich). The resulting solution was purified using Ni²^+^ affinity chromatography (Transgene). Protein refolding was achieved by dialyzing the purified protein against decreasing concentrations of urea until the urea was completely replaced by PBS. The recombinant SUMO protein was expressed as soluble form using pCold-SUMO vector and purified using Ni²^+^ affinity chromatography (Transgene, Beijing, China) as well. These recombinant proteins were finally maintained in PBS and quantified using a BCA Protein Assay Kit (Thermo Fisher Scientific).

### Immunofluorescence assay (IFA)

Cells were fixed at 7 days post-transfection of different HEV-RNAs by using 2% paraformaldehyde for 15 min at RT and then permeabilized with PBS containing 0.5% Triton X-100 (Sigma-Aldrich). Next, cells were probed with rabbit anti-p239 polyclonal antibodies and Alexa Fluor 555-labeled goat anti-rabbit IgG (Thermo Fisher Scientific), as well as indicated antibodies to visualize mitochondria. Cellular nuclei were counterstained with 4՛, 6-diamidino-2-phenylindole (DAPI; Thermo Fisher Scientific) at 37°C for 10 min. Images were captured under a Leica DM1000 fluorescence microscope (Leica Microsystems, Wetzlar, Germany) and processed using Leica Application Suite X (Version 1.0, Leica Microsystems). The co-localization of different fluorescence channels was analyzed using ImageJ software (Version 1.5.1, National Institutes of Health, Bethesda, MD, USA).

### RNA isolation and quantitative real-time PCR (qPCR)

Total RNA was extracted from cells or cell culture supernatant using TRIzol Reagent in accordance with the manufacturer’s instructions. Reverse transcription was conducted using a PrimeScript RT reagent Kit (TaKaRa, Dalian, China). Real-time PCR with 2× RealStar Power SYBR Mixture (Genstar, Beijing, China) as described previously (66). Transcripts for GAPDH were amplified from the same cDNA to normalize total RNA input. Gene expression was quantified by the 2^-∆∆^CT method (67). HEV RNA was detected using PerfectStart II Probe qPCR SuperMix (TransGene) following the manufacturer’s instructions. For absolute quantification of HEV-RNA copies, the infectious clone of HEV-3 KernowC1 p6 strain was used to generate a standard curve. Primers and corresponding DNA sequences were listed in Table 1.

### Statistical analysis

The results were analyzed using GraphPad Prism version 5.0 (GraphPad Software, San Diego, CA, USA). Statistical significance was determined using either the Student’s *t*-test for comparison of two groups or by one-way analysis of variance (ANOVA) for more than two groups. A two-tailed *P* value < 0.05 was considered statistically significant.

## Data Availability

The authors confirm that the data supporting the findings of this study are available and included within this article.
